# A “Pretender” Croconate-Bridged Macrocyclic Tetraruthenium Complex: Sizable Redox Potential Splittings despite Electronically Insulated Divinylphenylene Diruthenium Entities

**DOI:** 10.3390/molecules26175232

**Published:** 2021-08-29

**Authors:** Nils Rotthowe, Michael Linseis, Lars Vogelsang, Nicole Orth, Ivana Ivanović-Burmazović, Rainer F. Winter

**Affiliations:** 1 Fachbereich Chemie der Universität Konstanz, Universitätsstraße 31, 78464 Konstanz, Germany; nils.rotthowe@uni-konstanz.de (N.R.); michael.linseis@uni-konstanz.de (M.L.); lars.vogelsang@uni-konstanz.de (L.V.); 2 Department of Chemistry, University of Southern California, LJS 251, 840 Downey Way, Los Angeles, CA 90089, USA; 3 Department Chemie und Pharmazie, Friedrich-Alexander-Universität Erlangen-Nürnberg, Egerlandstraße 1, 91058 Erlangen, Germany; nicole.orth.no@gmail.com (N.O.); ivana.ivanovic-burmazovic@cup.uni-muenchen.de (I.I.-B.); 4 Department Chemie, Ludwigs-Maximilians-Universität München, Butenandtstr. 5-13, Haus D, 81377 München, Germany

**Keywords:** metallamacrocycle, electrochemistry, spectroelectrochemistry, crystal structure, redox splitting

## Abstract

Careful optimization of the reaction conditions provided access to the particularly small tetraruthenium macrocycle **^2^Ru_2_Ph-Croc**, which is composed out of two redox-active divinylphenylene-bridged diruthenium entities {Ru}-1,4-CH=CH-C_6_H_4_-CH=CH-{Ru} (Ru_2_Ph; {Ru} = Ru(CO)Cl(P*^i^*Pr_3_)_2_) and two likewise redox-active and potentially non-innocent croconate linkers. According to single X-ray diffraction analysis, the central cavity of **^2^Ru_2_Ph-Croc** is shielded by the bulky P*^i^*Pr_3_ ligands, which come into close contact. Cyclic voltammetry revealed two pairs of split anodic waves in the weakly ion pairing CH_2_Cl_2_/NBu_4_BAr^F^_24_ (BAr^F^_24_ = [B{C_6_H_3_(CF_3_)_2_-3,5}_4_]^−^ electrolyte, while the third and fourth waves fall together in CH_2_Cl_2_/NBu_4_PF_6_. The various oxidized forms were electrogenerated and scrutinized by IR and UV/Vis/NIR spectroscopy. This allowed us to assign the individual oxidations to the metal-organic Ru_2_Ph entities within **^2^Ru_2_Ph-Croc**, while the croconate ligands remain largely uninvolved. The lack of specific NIR bands that could be assigned to intervalence charge transfer (IVCT) in the mono- and trications indicates that these mixed-valent species are strictly charge-localized. **^2^Ru_2_Ph-Croc** is hence an exemplary case, where stepwise IR band shifts and quite sizable redox splittings between consecutive one-electron oxidations would, on first sight, point to electronic coupling, but are exclusively due to electrostatic and inductive effects. This makes **^2^Ru_2_Ph-Croc** a true “pretender”.

## 1. Introduction

Their inherent symmetry and the relative ease of their fabrication by the self-assembly of mononuclear metal-coligand nodes or dinuclear clamps and ditopic organic linkers as well as their structural adaptability render metallamacrocycles a particularly appealing class of compounds [1,2,3,4,5,6,7]. Besides their sheer beauty, their ability to undergo redox processes as encoded by the their metal constituents or the use of redox-active linkers has led to interesting applications that range from the redox-triggered release of incorporated guest molecules [8,9,10] to their use as superior water oxidation catalysts [11,12,13,14]. Some redox-active metallamacrocycles were also found to exhibit electronic coupling between individual subunits in their mixed-valent redox states via either through-bond or through-space pathways. Through-space interactions profit from face-to-face arrangements between planar, parallel disposed, redox-active, and π-conjugated organic ligands, as was masterfully demonstrated by Hupp and coworkers [15,16,17,18]. These authors employed ligand-bridged dirhenium clamps of variable extensions with one free binding site on every rhenium ion to bring two redox-active diimine linkers at the opposite sides of rectangular macrocyclic complexes in close spatial proximity, resembling the distance between individual sheets in graphite. The mixed-valent forms of these compounds, with one reduced and one neutral diimine ligand, are electronically coupled with a coupling strength that depends on their spacing [15,16,17,18]. Through-bond coupling in metallamacrocyclic architectures was first indicated by sizable redox splittings between individual oxi-dations in trinuclear macrocyclic 3-hydroxypyridinone-bridged half-sandwich ruthenium complexes [19], and later demonstrated for related adenine-bridged congeners with Ru^II^([9]aneS_3_) metal nodes ([9]aneS_3_ = 1,4,7-trithiacyclononane) [20,21]. We have more recently reported similar phenomena in macrocyclic tri- or tetraruthenium complexes constructed from self-complimentary {Ru(CO)(P*^i^*Pr_3_)_2_(OOC-Arylene-CH=CH-)} building blocks derived from 3-ethynylbenzoic acid or 5-ethynylthiophene-2-carboxylic acid. The occupied frontier orbitals of such repeat units spread over the entire Ru-CH=CH-Aryl entity [22,23,24], while the carboxylate linker represents a local node, thereby attenuating electronic coupling in the mixed-valent states [25,26]. In contrast, structurally similar macrocyclic ruthenium complexes built from {Ru}-CH=CH-Aryl-CH=CH-{Ru} entities, capable of forming fully delocalized mixed-valent radical cations, and isophthalate, terephthalate, isonicotinate or pyrrole-, furan- or thiophene-2,5-dicarboxylate linkers do not exhibit any signs of electronic coupling between the π-conjugated diruthenium entities, as the dicarboxylate linkers do not contribute to the relevant redox orbitals [25,27,28,29,30,31,32].

**Figure 1:**
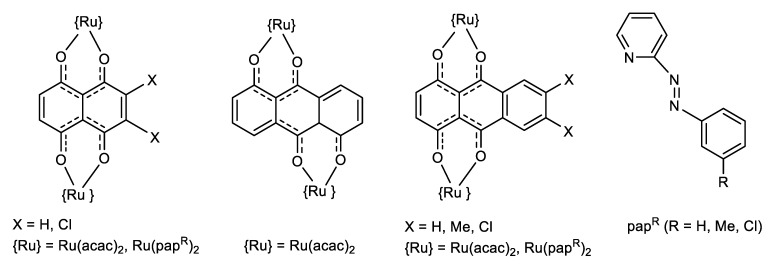
Examples of diruthenium complexes with bis(β-ketoenolate) bridging ligands capable of electronic coupling in the one-electron oxidized states.

One possible approach to overcoming this “segregation” problem is to utilize redox-active bridging ligands with similarly high-lying occupied frontier molecular orbitals (FMOs) as the alkenyl ruthenium entities. Such “non-innocent” ligands [33,34,35] may hence participate in anodic redox processes and exhibit low activation energies for interligand, ligand-metal, or metal-ligand charge transfers. Viable candidates can be found amongst aromatic bis(β-ketoenolates) such as 5,8-dioxido-1,4-anthraquinones or 1,4-dioxido-9,10-anthraquinones. Representatives of this ligand class and their amino-/imino congeners have a demonstrated ability to foster electronic coupling between two interlinked, electron-rich ruthenium complex entities and/or to actively contribute or even dominate redox processes, thus leading to variable and often intricate charge state distributions [36,37,38,39,40,41]. Some examples of such complexes are portrayed in Figure 1. The ligands of Figure 1, however, do not lend themselves for constructing metallamacrocycles with {Ru}-CH=CH-Aryl-CH=CH-{Ru} building blocks, owing to the linear disposition and the close spatial proximity of the two β-ketoenolate binding pockets. This does not allow them to accommodate two bulky, electron-rich Ru(P*^i^*Pr_3_)_2_ fragments, which are otherwise highly advantageous to stabilize the oxidized forms of such complexes.

**Figure 2:**
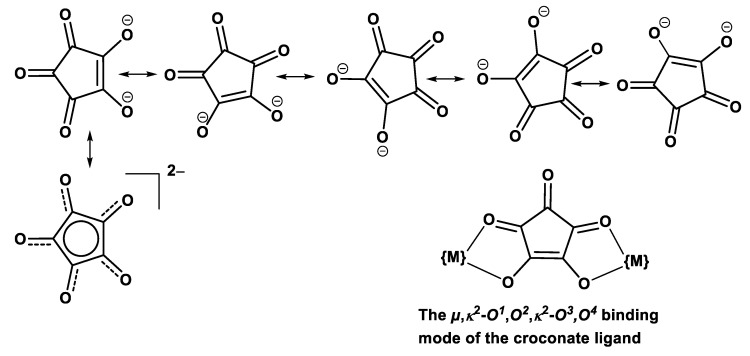
Resonance structures for the croconate dianion and its envisioned bis(chelating) binding mode for providing metallamacrocyclic structures.

In our search of π-conjugated bis(β-ketoenolate) ligands with an angular arrangement of the chelating functionalities, we identified the croconate dianion C_5_O_5_^2−^ as a particularly promising candidate. Croconate belongs to the compound class of oxycarbons with the general formula C_n_O_n_^m−^ and was first synthesized as early as in 1825 [42]. Its name derives from the yellow color of its metal salts, which is highly reminiscent of some variants of the crocus flower and of egg yolk. With its planar structure, its (nearly) perfect fivefold symmetry, even in the crystalline state [43,44,45], and its two delocalized π-electrons, the croconate dianion meets the formal criteria of a Hückel arene (Figure 2). Quantum chemical studies however indicate that its degree of aromaticity is considerably lower than that of ordinary arenes [44,46]. Moreover, the croconate dianion undergoes two reversible one-electron oxidations at similarly low potentials as divinylarylene-bridged diruthenium complexes [23,47,48,49,50,51,52] (e.g., E_1/2_^2−/−^ = −240/−310 mV, E_1/2_^−/0^ = 5/100 mV against the ferrocene/ferrocenium redox couple FcH/FcH^+^ in DMF as the solvent, depending on the supporting electrolyte) [53,54]. Electroactivity is usually also maintained on complexation with concomitant shifts to more anodic potentials as a result of electron donation to the metal ions [55].

Among the many coordination modes that this highly adaptive ligand may exhibit in transition metal complexes [56,57], the *µ*,*κ*^2^*-O*^1^,*O*^2^,*κ*^2^*-O*^3^,*O*^4^-binding mode (Figure 2), which has been observed on several occasions [56,57,58,59,60,61,62], would be well-suited to forge divinylphenylene-bridged diruthenium building blocks {Ru(CO)(P*^i^*Pr_3_)_2_}_2_(1,4-CH=CH-C_6_H_4_-CH=CH), henceforth abbreviated as Ru_2_Ph, into macrocyclic structures. In addition, croconate also offers the degree of coordinative lability and flexibility of its coordination modes required to transform initially formed mixtures of different di- or oligomeric complexes into the thermodynamically preferred macrocycle(s) [30,31,63,64,65,66,67]. Here, we report on the successful isolation and in-depth characterization of a tetranuclear macrocyclic complex with considerably smaller dimensions as previous dicarboxylate-bridged congeners. Of particular relevance is the observation of three or even four individually resolved one-electron oxidations. The issues of the loci of the anodic processes and of the presence or absence of electronic coupling in the mixed-valent states are addressed by a multimethod approach, including IR, UV/Vis/NIR, and EPR spectroscopy with support by quantum chemistry.

## 2. Results and Discussion

### 2.1. Synthesis, Spectroscopic Identification, and X-ray Crystallography

**Scheme 1:**
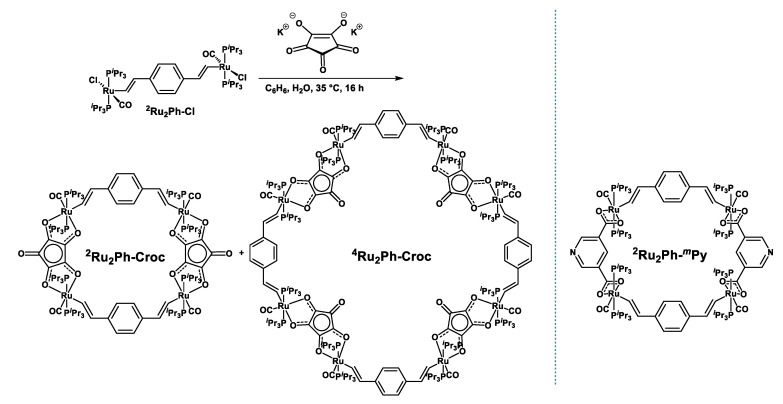
(**left**): Synthesis and putative structures of the new macrocyclic complexes **^2^Ru_2_Ph-Croc** and **^4^Ru_2_Ph-Croc**; (**right**): the isonicotinate-bridged tetraruthenium macrocycle **^2^Ru_2_Ph-*^m^*Py**.

**Figure 3:**
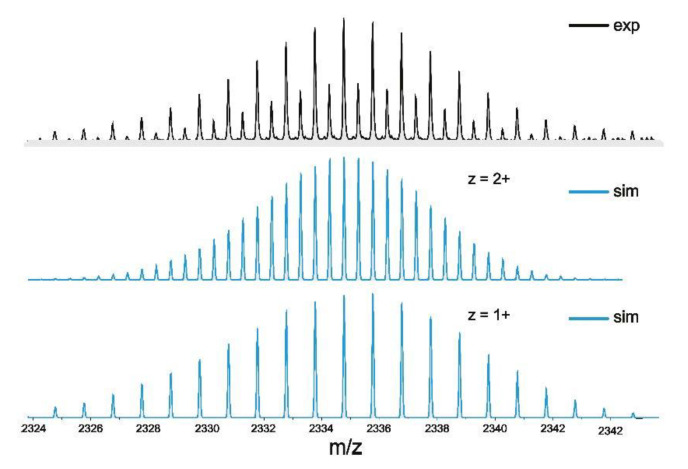
High-resolution ESI mass spectrum of the initial product mixture of tetranuclear **^2^Ru_2_Ph-Croc** and octanuclear **^4^Ru_2_Ph-Croc** obtained from the reaction of **Ru_2_Ph-Cl** with potassium croco-nate (**top**). Simulated spectrum of **^4^Ru_2_Ph-Croc^2+^** (**middle**) and of **^2^Ru_2_Ph-Croc^+^ (bottom**).

The insolubility of alkali metal salts of croconic acid in methanol called for the use of biphasic mixtures consisting of a solution of the 1,4-divinylphenylene-bridged diruthenium precursor {Ru(CO)Cl(P*^i^*Pr_3_)_2_}_2_(1,4-CH=CH-C_6_H_4_-CH=CH) (**Ru_2_Ph-Cl**) [47] in either CH_2_Cl_2_ or benzene, an aqueous solution of a slight excess (1.06 equivalents) of potassium croconate monohydrate [68,69], and a few drops of methanol as a phase transfer agent for synthesis. NMR spectra obtained from the solid product obtained after appropriate workup (see the Experimental Section) indicated the presence of two different species as revealed by the two sharp singlet resonances in the ^31^P NMR spectrum and two sets of resonances for the Ru-C*H*=CH and the Ru-CH=C*H* protons with integration ratios of ca. 3:2 (Scheme 1 and Figures S1 and S2 of the Supplementary Materials). This pattern is suggestive of two kinds of macrocyclic complexes that differ in their nuclearities, similar to what we have observed on previous occasions [27,30,31]. High-resolution mass spectro-metry indeed provides a molar peak at 2336.78 g mol^−1^ (calcd. 2336.77) which comprises of two different envelopes, one giving rise to an isotopic pattern with spacings by one mass unit, and one with a half-integer spacing (Figure 3). The observed pattern thus corresponds to the molecule ion peak of a tetranuclear species **^2^Ru_2_Ph-Croc** that consists of two equivalents of each building block, and that of the doubly charged octanuclear complex **^4^Ru_2_Ph-Croc** comprising of four equivalents of each constituent. Putative structures of these supramolecular isomers are shown in Scheme 1. They most likely differ with respect to the orientation of the croconate ligand, i.e., by whether the non-coordinating carbonyl functionality is oriented outwards (**^2^Ru_2_Ph-Croc**) or towards the interior (**^4^Ru_2_Ph-Croc**) of the macrocyclic cavity and by the ensuing angles of the Ru–C_5_O_5,centre_–Ru vector of ca. 72° or 144° as dictated by the fivefold symmetry of the croconate dianion. All attempts to separate the two differently sized macrocycles through either enrichment by repeated washings, fractionated crystallization or selective precipitation remained futile. In consequence, the reaction conditions had to be optimized in such a way as to favor the formation of one kind of macrocycle over the other (note that chromatographic separation is not possible to the sensitivity of the Ru-vinyl linkage to any Brønsted or Lewis acid).

Through an iterative process of varying reaction time, temperature, and solvent we were able to identify conditions that afforded pure **^2^Ru_2_Ph-Croc**, namely: (i) using a saturated (27 mM) solution of **Ru_2_Ph-Cl** in benzene as the tetranuclear macrocycle is less soluble in this solvent than the octanuclear congener and hence precipitates first; (ii) conducting the reaction at a slightly elevated reaction temperature of 35 °C; and (iii) limiting the reaction time to 19 h, as the smaller macrocycle constitutes the kinetic product. Shorter reaction times led to incomplete conversion of difficult-to-separate **Ru_2_Ph-Cl**, while longer reaction times lead to increasing amounts of the larger macrocycle. **^2^Ru_2_Ph-Croc** could be ultimately obtained in a yield of 21%, which is only modest when compared to what can be achieved for similar dicarboxylate-linked macrocycles [25,28,29,30,31,32]. Underlying reasons are that a significant portion of the tetranuclear macrocycle remains in solution and cannot be separated from slightly better soluble **^4^Ru_2_Ph-Croc** and the difficulties encountered when separating the precipitated solid from the biphasic supernatant.

**Figure 4:**
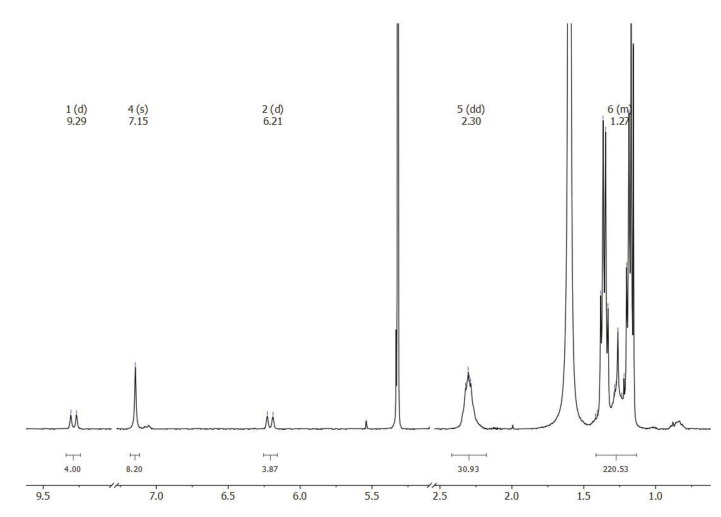
^1^H-NMR spectrum of **^2^Ru_2_Ph-Croc** in CD_2_Cl_2_.

The high purity of isolated **^2^Ru_2_Ph-Croc** is evident from the ^1^H-, ^13^C-, and ^31^P-NMR spectra (see Figure 4 and S3–S5 of the Supplementary Materials) as well as by mass spectrometry (see Figure S6 of the Supplementary Materials). The simplicity of the NMR patterns pay witness to the high symmetry of **^2^Ru_2_Ph-Croc**, with only one doublet resonance for each of the vinylic Ru-C*H* and the Ru-CH=C*H* protons at 9.29 and 6.21 ppm, respectively (^3^*J*_HH_ = 15.7 Hz), one singlet resonance for the phenylene protons at 7.15 ppm, and the expected resonances for the C*H* and the CH(C*H*_3_)_2_ protons of the P*^i^*Pr_3_ ligands at 2.30 (C*H*(CH_3_)_2_) or 1.37 and 1.15 ppm for the diastereotopic methyl groups. Of note is the shift of the Ru-C*H* resonance to unusually low field as compared to ca. 8.5 ppm in dicarboxylate-bridged tetraruthenium macrocycles and to 8.87 ppm in the larger **^4^Ru_2_Ph-Croc**. This points to a large torsion of the Ru-CH=CH entities with respect to the plane of the phenylene linker and a concomitantly smaller degree of π-conjugation. The reduction of the symmetry of the bridging C_5_O_5_^2−^ ligand from fivefold rotational symmetry to only a mirror plane through the uncoordinated keto function and the midpoint of the opposite C–C bond is mirrored by the observation of three distinct ^13^C NMR resonances at 199.1, 184.8, and 180.6 ppm for the ring carbon atoms.

**Figure 5:**
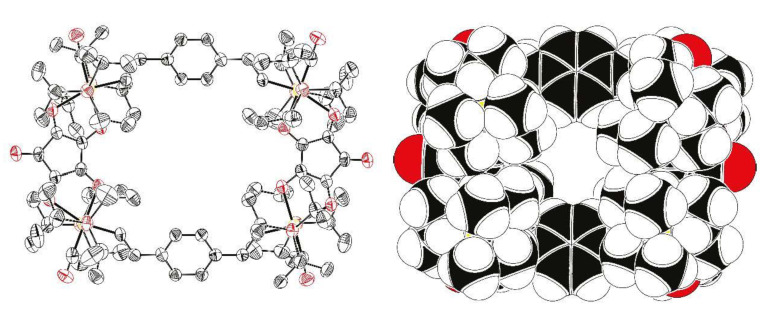
ORTEP representation (**left**) and space-filling model (**right**) of the molecular structure of **^2^Ru_2_Ph-Croc** in the single crystal. Thermal ellipsoids are displayed at 50% probability. Hydrogen and solvate atoms are omitted for clarity reasons.

Slow diffusion of methanol into a dichloromethane solution containing **^2^Ru_2_Ph-Croc** yielded a small crop of crystals that proved suitable for single crystal X-ray crystallo-graphy. Crystallographic details and a list of bond lengths and angles are provided in the Supplementary Materials (Tables S1–S3 and Figure S8). Figure 5 displays the molecular structure.

**^2^Ru_2_Ph-Croc** crystallized in the monoclinic space group *P*12_1_/*n*1 along with two CH_2_Cl_2_ and one MeOH solvate molecules per formula unit. The latter are disordered over three (CH_2_Cl_2_) or four sites (MeOH) and were treated by the SQUEEZE implementation [70] of OLEX [71]. The structure is inversion symmetric, rendering diagonally disposed vinyl ruthenium fragments equivalent. Assuming that the maximum accessible cavity has the shape of an octagon spanned by the coordinated oxygen atoms of the croconate ligand pointing inside the void and the inwards orientated hydrogen atoms on the phenylene linkers, one obtains an area of 55.2 Å^2^ with dimensions of 9.87 Å × 7.00 Å for its long (O···O distance) and short (H···H distance) axis. This equals to just about 50% to 60% of the size of the rectangular cavity in the benzoate-bridged analog [{Ru(CO)(P*^i^*Pr_3_)_2_}_2_(µ-1,4-CH=CH-C_6_H_4_-CH=CH)]_2_{1,3-(OOC)_2_-C_6_H_3_-5-SAcyl}_2_ [29]. The decreased dimensions of the void are the result of the much smaller Ru···Ru distance of only 7.25 Å across the croconate linker as compared to 9.28 Å in the dicarboxylate derivative. The small size of the macrocycle leads to a close approach of the sterically demanding P*^i^*Pr_3_ ligands. Hence, the closest H···H distances of 2.48 Å are only marginally larger than the contact Van-der-Waals distance of 2.40 Å. This not only suggests that the phosphine ligands act as umbrellas, but also that the croconate dianion is potentially one of the smallest bis(chelate)s capable of linking two Ru(CO)(P*^i^*Pr_3_)_2_(CH=CH-R) fragments. The close approach of the Ru(P*^i^*Pr_3_)_2_ moieties also leads to an increase of the Ru-CH=CH angles to 138.1(4) or 140.4(4)° compared to the 129.7(16) and 133.4(14)° found in the 4-thioacetylisophthalate-linked congener [29], as well as a sizable torsion of 28.6° of the Ru-CH=CH entities out of the plane of the phenylene linker to relieve steric strain between H_α_ and the hydrogen atoms of the phenylene unit. This matches with the unusual low-field shift of the H_α_ resonance in the ^1^H-NMR spectrum (vide supra), indicating that the relatively large torsion is also retained in solution.

The five-membered ring formed by the croconate chelate and the ruthenium ion imposes an only modest distortion of the octahedral coordination geometry with O-Ru-O angles of 75.59(13)° and 76.20(13)°, respectively. This is to be compared to 59.1(4)° and 58.5(4)° in the four-membered chelate rings formed by the carboxylates in the 4-thioacetylisophthalate complex. Ruthenium coordination also localizes the C=C and C=O double bonds within the croconate ligand as is evident from the inequivalence of all C-C and C=O bonds. The non-coordinating carbonyl function is associated with the shortest C=O bond length of 1.229(6) Å, while the other CO bonds of 1.248(6) to 1.285(6) Å are appreciably longer. Likewise, the C3-C6 and C5-C6 bonds to the uncoordinated carbonyl group of the croconate ligand of 1.498(8) and 1.489(7) Å are longer than the remaining ones of 1.414(8), 1.446(7) and 1.457(7) Å, indicating that delocalization of the C=C double bond is confined to the two ketoenolate-type binding pockets (note that the C-C bonds in the dipotassium dihydrate salt are 1.465(3) and 1.474(3) Å) [72].

### 2.2. Electrochemistry

**Figure 6:**
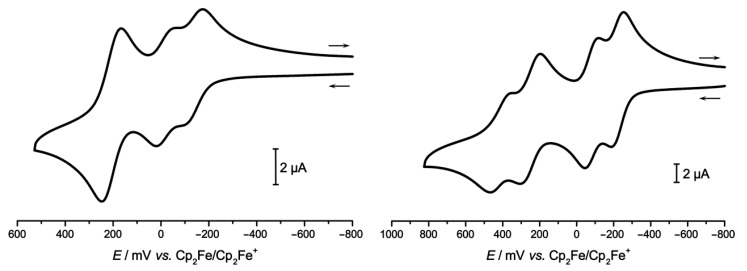
Anodic scans in the cyclic voltammograms of **^2^Ru_2_Ph-Croc** in CH_2_Cl_2_ with either 0.1 M NBu_4_PF_6_ (**left panel**) or NBu_4_BAr^F^_24_ (**right panel**) as the supporting electrolyte.

The electrochemical behavior of **^2^Ru_2_Ph-Croc** was probed in CH_2_Cl_2_ as the solvent and was found to depend on the ion-pairing capabilities of the counter ion of the suppor-ting electrolyte. With NBu_4_PF_6_, three anodic redox events can be observed at *E*_1/2_ values of −134 mV, −17 mV and 207 mV. As is evident from Figure 6, the first two waves correspond to one-electron oxidations to the respective mono- and dication, whereas the third event entails two electrons and hence corresponds to the further charging to the tetracation. The use of NBu_4_BAr^F^_24_ as the supporting electrolyte (BAr^F^_24_^−^ = [B{C_6_H_3_(CF_3_-3,5)_2_}_4_]^−^) also resolves the third and fourth oxidations into separate one-electron processes and also increases the potential separations between the 0/+//+/2+ waves. The corresponding data are compiled in Table 1 along with those of the **Ru_2_Ph-Cl** precursor and the analogous iso-nicotinate-bridged tetraruthenium congener **^2^Ru_2_Ph-*^m^*Py** of Scheme 1.

Similar responses, albeit with considerably lower redox splittings between the merged one-electron waves of the 0/2+ and 2+/4+ processes, were observed for other macrocyclic tetraruthenium complexes comprising of π-conjugated divinylarylene diruthenium building blocks. This pattern indicates the nearly coincident charging of the opposite {Ru}-CH=CH-Aryl-CH=CH-{Ru} electrophores by first one and then a second electron. The only small or vanishing potential splitting between the 0/+ and +/2+ or the 2+/3+ and 3+/4+ waves reflects electrostatic interactions between them, as the insulating dicarboxylate linkers prevent through-bond electronic coupling [27,28,29,30,31]. In this particular case, however, an alternative scenario, where one of these composite waves originates from the first oxidations of opposite croconate linkers, is also conceivable, such that the loci of the individual oxidations remain to be verified. One should note here that neutral C_5_O_5_ is only stable in water, forming leuconic acid, i.e., the pentaketone pentahydrate C_5_O_5_·5H_2_O, whereas in other solvents the two-electron oxidized form is chemically unstable [54,68]. In spite of the presence of two kinds of electroactive building blocks, which are both capable of losing two electrons each, no further charging processes could be observed within the potential windows of the used electrolytes.

Irrespective of the identity of the oxidation sites, the use of a croconate instead of a dicarboxylate bis(chelate) increases the thermodynamic stabilities of the mixed-valent forms as evident from the enhanced Δ*E*_1/2_ values between the 0/+ and +/2+, or the 2+/3+ and the 3+/4+ redox couples. While the sizable effects of the anion of the supporting electrolyte point towards a significant contribution of electrostatic effects to the observed redox splittings, electrochemical studies per se are inconclusive as to whether this increase in redox splitting also entails contributions from improved electron delocalization [73,74,75,76,77,78,79]. The presence or absence of electronic coupling can, however, be probed by combining (electro)chemical oxidation with IR and UV/Vis/NIR spectroscopy, and this is the subject of the following section.

In passing we add that cathodic scans reveal two closely spaced reductions associated with the croconate linkers as chemically only modestly reversible waves with half-wave potentials of −1930 mV and −2010 mV. In NBu_4_BAr^F^_24_ the reduction can just be made out at the cathodic limit of the potential window as highly overlapped peaks with an associated average *E*_1/2_^0/−^ of −2000 mV as determined through square-wave voltammetry. Graphical accounts of these waves can be found in the Supplementary Materials (Figure S9). These processes are of no concern in the present context and are not further considered.

### 2.3. IR and UV/Vis/NIR Spectra of the Oxidized Forms and Insights from Quantum Chemistry

As was discussed in the previous section, molecular spectroscopy of the oxidized forms was conducted in order to provide answers to the open questions about the identity of the electron transfer sites in **^2^Ru_2_Ph-Croc** and on the existence and strength of any electronic coupling in the mixed-valent states. As to the first issue, the carbonyl ligands at the ruthenium ions and the characteristic carbonyl and CC stretches of the bridging ligands of **^2^Ru_2_Ph-Croc** offer convenient IR labels that respond to changing electron densities at these local sites as a result of the stepwise oxidations. While the spectroscopic responses expected of Ru_2_Ph-based redox processes are amply known, we also conducted similar studies on (NBu_4_^+^)_2_ croc^2^^−^ [80], which is soluble in CH_2_Cl_2_. Due to the inability of CH_2_Cl_2_ to form strong hydrogen bonds, only the first oxidation is reversible (see Figure S10 of the Supplementary Materials) [54]. Accompanying spectroscopic changes in the IR spectrum are displayed in Figure S11 of the Supplementary Materials and entail the bleaching of the strong band at 1520 cm^−1^ and the emergence of a new, weaker band at 1552 cm^−1^ as the most characteristic changes.

**Figure 7:**
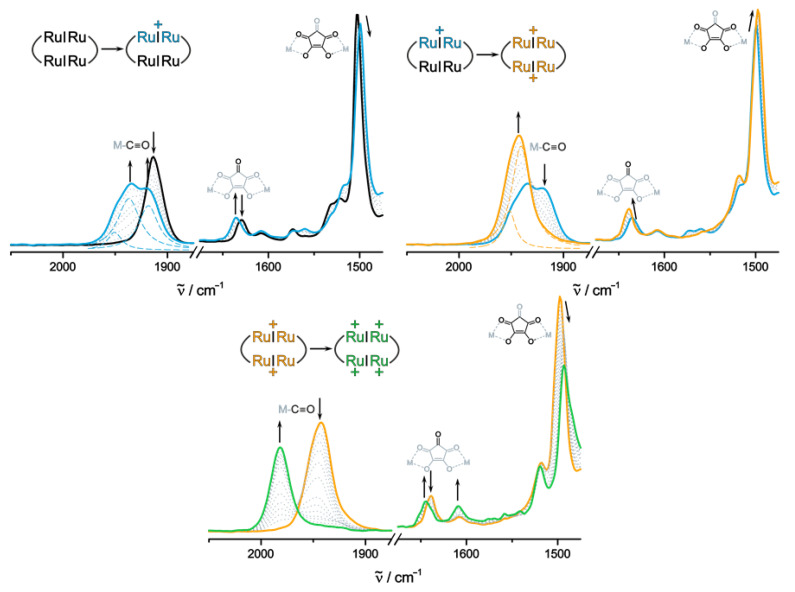
Changes of the IR spectra (DCE, 0.05 M NBu_4_BAr^F^_24_, r.t.) of **^2^Ru_2_Ph-Croc** in the Ru(CO) and the region of the ligand-based C=O/C=C stretching vibrations during the oxidation to the monocation (**top left**), dication (**top right**), and the tetracation (**bottom**). Deconvolution of the Ru(CO) bands was performed where necessary (dashed colored lines).

The various oxidized forms **^2^Ru_2_Ph-Croc^n+^** (n = 1–4) were electrogenerated inside a thin-layer electrolysis cell [81] by increasing the applied working potential in increments until a new equilibrium had established. Owing to the enhanced redox splittings and the higher propensity for also observing the mixed-valent states without interference from the bordering isovalent states, we conducted these experiments on solutions of the complex in 0.05 M NBu_4_BAr^F^_24_ in the less volatile 1,2-dichloroethane (DCE) as the solvent. Graphical accounts of the outcomes of such experiments are displayed in Figure 7, while pertinent data are summarized in Table 2. The IR spectra of **^2^Ru_2_Ph-Croc** in the neutral as well as cationic states in the full range between 1000 cm^−1^ and 4000 cm^−1^ are shown in Figure S12 of the Supplementary Materials.

In its neutral state, **^2^Ru_2_Ph-Croc** shows a single CO band for the ruthenium-bonded carbonyl ligands Ru(CO) at 1913 cm^−1^ (see top left graph of Figure 7). Despite the formally higher number of 18 valence electrons at each {Ru} fragment in **^2^Ru_2_Ph-Croc**, the absolute values for the Ru(CO) stretches match those of parent **Ru_2_Ph-Cl**, where the ruthenium ions attain a lower electron count of only 16 valence electrons. This contrasts sharply to a sizable red-shift by 13 cm^−1^ on introducing an acac^−^ donor [82]. This difference is likely rooted in the combined effects of the limited electron donating capabilities of the croco-nate bridging ligand and attenuated π-conjugation along the divinylphenylene backbone due to the rather sizable torsion at the vinyl-phenylene linkages (vide supra). The position and overall shape of this band are fully reproduced by our quantum chemical calculations. The latter predict two nearly coincident peaks for the symmetric and antisymmetric stretches of the carbonyl ligands on two diagonally related {Ru} entities at 1913 and 1914 cm^−1^. During the first oxidation an intricate Ru(CO) band pattern emerges, which could be deconvoluted into three distinct bands (see blue dashed lines in the top left graph of Figure 7). Such a three-band pattern is also predicted by the quantum chemical vibrational analysis with features at $\tilde{\nu }$_calc_ = 1920, 1934 and 1942 cm^−1^, in excellent agreement with the experiment. Based on the calculations and chemical intuition, the band found at 1917 cm^−1^ can be assigned to the carbonyl stretch of the remaining neutral Ru_2_Ph unit. The remaining two bands at 1935 and 1949 cm^−1^ correspond to the less intense symmetric and the intense antisymmetric combinations of Ru(CO) stretches of the one-electron oxidized divinylphenylene diruthenium moiety. The overall Ru(CO) band shifts and the increased energy difference between the two vibrational modes of the oxidized divinylphenylene diruthenium entity are in complete agreement with the oxidation-induced changes for other, related complexes of this type [49,50,51,52,83,84]. During the second oxidation, the band at 1917 cm^−1^ starts to fade, while the other two bands shift further blue, to 1941 and 1952 cm^−1^. The pattern of one intense and one less intense Ru(CO) band is a clear indicator that the di-cation **^2^Ru_2_Ph-Croc^2+^** is comprised of two singly oxidized Ru_2_Ph^•^^+^ entities. A further argument against croconate-based oxidations is provided by the very modest red/blue shifts of the croconate CO bands corresponding to the chelating (1503 cm^−^^1^ → 1499 cm^−^^1^ → 1498 cm^−^^1^) and the uncoordinated keto CO functions (1630 cm^−^^1^ → 1636 cm^−^^1^ → 1639 cm^−^^1^). The experimental band pattern is well matched by the computed Ru(CO) vibrational modes at $\tilde{\nu }$_calc_ = 1939, 1939, 1945 and 1948 cm^−1^ based on an input structure in the electronic triplet state. We note here that the data for the singlet form are very similar (see Table 2). The latter is by a computed margin of 60 kJ/mol higher in energy and its calculated spectros-copic features agree less well with our experimental data (vide infra). The mixed-valent form **^2^Ru_2_Ph-Croc^•^^+^** is thus characterized by a pattern of bands corresponding to the neutral and a double Ru(CO)-band-feature for the oxidized Ru_2_Ph subunits. The blue shift of the Ru_2_Ph subunit of **^2^Ru_2_Ph-Croc^+^** by 3 cm^−^^1^ and the (averaged) red-shift of the Ru_2_Ph^•^^+^ entity of 4 cm^−1^ with respect to the bordering isovalent states **^2^Ru_2_Ph-Croc** and **^2^Ru_2_Ph-Croc^2+^** are very modest and may well be entirely due to inductive effects transmitted via the croconate linkers [82,85].

**Figure 8:**
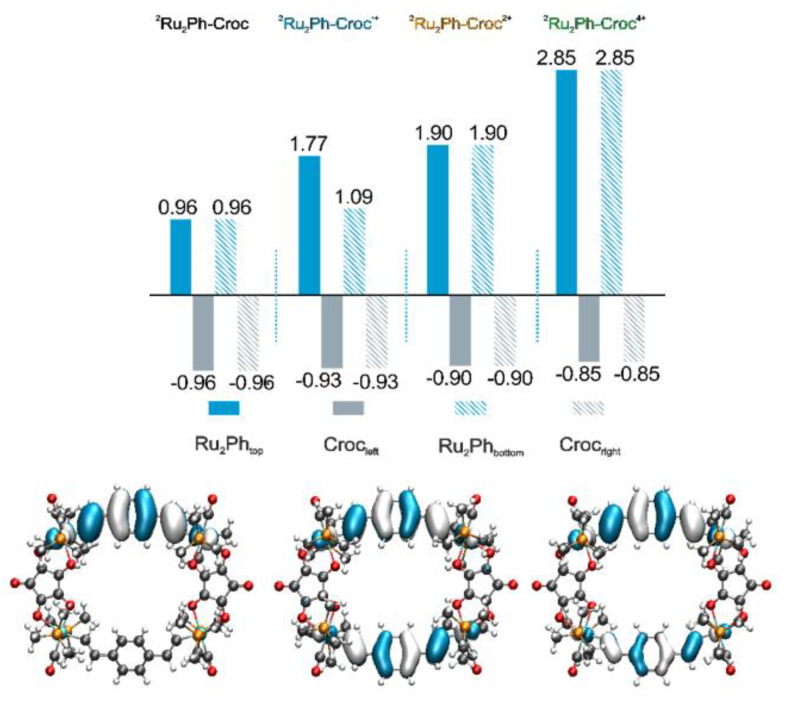
Charge densities on different constituents of **^2^Ru_2_Ph-Croc** in its neutral, monocationic, dicationic, and tetracationic states according to NBO analysis (**top**) and contour plot of the β-LUSO of the monocation (**bottom left**), the dication (**bottom middle**) and of the LUMO in the tetracation (**bottom right**). The notations **top**, **bottom**, **left,** and **right** correspond to the complex entities as they are depicted in the bottom panel.

In line with the above presumption, further oxidation did not provide any indication for a unique spectroscopic signature of a tricationic form, either in the IR nor the NIR region of the spectrum. This implies that the triacation has no specific spectroscopic fingerprint distinguishing it from mixtures of the di- and the tetracation. Hence, only the disappearance of the bands associated to the monocationic Ru_2_Ph^•^^+^ fragments with concomitant formation of a new Ru(CO) band at 1982 cm^−1^ were observed. The presence of only a single, shifted Ru(CO) band is a clear indicator that the second pair of oxidations also involves the Ru_2_Ph entities and that all four {Ru} nodes are electronically equivalent. Again, the shift of vibrational bands associated with the croconate ligands are modest (1639 cm^−1^ → 1644 cm^−1^; 1498 cm^−1^ → 1493 cm^−1^), which argues against any substantial involvement into also the higher oxidations. The computed energies of the carbonyl stretches agree well with the experimental data, regardless of the assumed spin state. Based on the results for dications of other divinylphenylene diruthenium complexes or their respective forms when arranged in a macrocycle, the singlet state should dominate [27,86]. The experimental finding of hardly any involvement of the croconate ligands to the individual redox processes is fully supported by our natural bond order (NBO) analysis (Figure 8). According to our calculations, the first two oxidations result in a charge loss of only 7% from the croconate ligands, with an additional 6% loss during the last two-electron charging process.

Based on the experimental results and in full agreement with our computations, it can be said that the mixed-valent mono- and trioxidized forms are best described as containing one neutral Ru_2_Ph/singly oxidized Ru_2_Ph^•^^+^ and one singly oxidized Ru_2_Ph^•^^+^/doubly oxidized Ru_2_Ph^2+^ entity. The accessibility and unique spectroscopic features of the moncation are thus likely the result of electrostatic repulsion caused by the close spatial proximity of the two redox-active divinylphenylene diruthenium fragments and to only a minor degree (if any at all) to electronic coupling across the croconate ligands.

**Figure 9:**
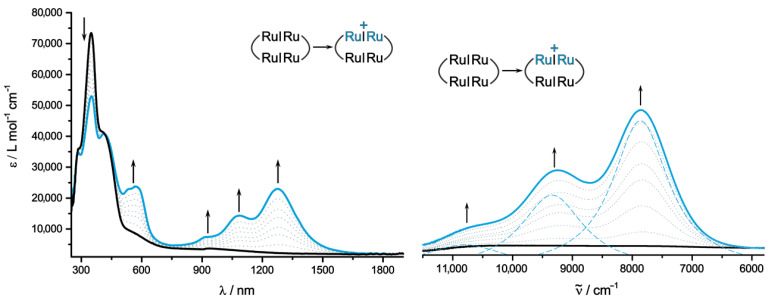
Changes in the UV/Vis/NIR spectrum (DCE, 0.05 M NBu_4_BAr^F^_24_, r.t.) of **^2^Ru_2_Ph-Croc** during the first oxidation (**left**); magnification of the IR/NIR part of the spectrum (**right**). The broken blue lines denote the positions of the individual sub-bands according to spectral deconvolution.

This issue of electronic coupling in the mixed-valent states is, however, best probed by electronic spectroscopy in the near infrared (NIR). All redox states that were accessible in the IR spectroelectrochemical experiments could also be interrogated by UV/Vis/NIR spectroscopy. Corresponding data derived from our experiments and from time-depen-dent density functional theory (TD-DFT) calculations are collected in Table 3. Our TD-DFT protocols match those of other authors that have proven successful for assessing the optical properties of organic dyes and metal complexes [87,88,89]. The electronic spectrum of neutral **^2^Ru_2_Ph-Croc** and its change during the first oxidation to the radical cation are depicted in Figure 9.

In contrast to other dicarboxylate-based macrocycles, **^2^Ru_2_Ph-Croc** is not faint yellow in color, but brown. This is due to the presence of a broad absorption shoulder near 550 nm. The underlying excitations, computed at λ_calc_ = 608 to 626 nm, entail charge transfer (CT) from the Ru_2_Ph moieties to the oxocarbon bridging ligands (see Figures S13–S16 in the Supplementary Materials for plots of the MOs involved in the transitions and electron density difference maps). Revealingly, the two highest occupied MOs as well as the two lowest unoccupied MOs are doubly degenerate and represent the in- and out-of-phase combinations of orbitals localized at the oppositely disposed Ru_2_Ph entities (HOMO, HOMO−1) or on the coroconate ligands (LUMO, LUMO+1). This degeneracy is a token for the lack of electronic interactions between the individual donor or acceptor fragments within the macrocyclic structure [83,90]; for an example of a π-conjugated organic macrocycle with non-degenerate FMOs and electronically coupled mixed-valent states see Ref. [91]. A shoulder at λ_max_ = 410 nm corresponds to the HOMO−2 to LUMO excitation and is hence characterized as a croconate-based π-π* transition with a calculated wavelength λ_calc_ of 431 nm. The most intense band at λ_max_ = 350 nm (λ_calc_ = 329 nm) corresponds to the π-π* transition on the divinylphenylene diruthenium (Ru_2_Ph) fragments. 

The most characteristic feature of the spectrum of **^2^Ru_2_Ph-Croc^•+^** are the three bands in the near infrared (NIR) at 1275 nm (7830 cm^−1^), 1085 nm (9300 cm^−1^), and 930 nm (10770 cm^−1^) in the order of decreasing absorbance (see blue broken lines in Figure 9, left). Furthermore, the Vis band at ca. 550 nm intensifies without any discernible shift, while the UV band originating from the Ru_2_Ph chromophores decreases in intensity. The rich structuration of the NIR absorption is characteristic for the radical cation of divinylphenylene-bridged diruthenium complexes and is traced back to vibrational coupling [47,86]. The strong similarities in the NIR absorbance between the radical cation **^2^Ru_2_Ph-Croc^•+^** and those of other tetranuclear metallamacrocycles with dicarboxylate linkers and the absence of any further absorption at lower energies, as would be expected in the case of intervalence charge transfer (IVCT) between the differently charged Ru_2_Ph subunits, equally suggest that the croconate bridges remain uninvolved in the oxidation and act as insulators. 

The TD-DFT calculations paint a somewhat different picture. They assign the pro-minent feature at λ_calc_ = 1011 nm to a transition, which involves charge transfer from a combination of orbitals that spread over both croconate bridging ligands and the remai-ning reduced divinylphenylene diruthenium building block (mainly the β-HOSO-1, see the Supplementary Materials). Judging by the TD-DFT results, this band is hence assigned as a mixed oxocarbon to Ru_2_Ph**^•+^** charge transfer (L-L’MCT) and IVCT rather than being confined to the oxidized divinylphenylene diruthenium unit. This seems, however, unlikely for the mentioned reasons and the alterations in the IR/NIR spectra imposed by further oxidation to the dication (vide infra). It thus appears as if the quantum chemical calculations overestimate the strength of adiabatic electronic coupling, which is a common problem of DFT-based methods [92,93]. TD-DFT also predicts an additional band at λ_max_ = 2171 nm, albeit with a very small oscillator strength, which has all characteristics of a classical IVCT transition. It is thus directed from the reduced to the singly oxidized divinylphenylene diruthenium entity. No such feature was, however, experimentally observed. The overestimated computed electron delocalization only affects the transitions in the NIR. All other bands in the Vis or the UV are adequately represented. Thus, the split band at λ_max_ = 570 and 535 nm corresponds to predicted transitions at λ_calc_ = 663 and 505 nm. They both entail CT from one of the Ru_2_Ph entities to the croconate linkers, with the excitation involving the oxidized Ru_2_Ph**^•+^** subunits at the higher energy. The π-π* transitions confined to the oxocarbon bridges as well as that on the reduced Ru_2_Ph entity remain basically unaltered during the first oxidation.

**Figure 10:**
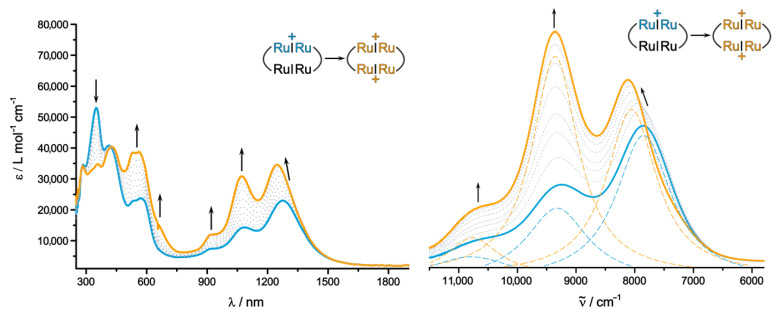
Changes in the UV/Vis/NIR spectrum (DCE, 0.05 M NBu_4_BAr^F^_24_, r.t.) of **^2^Ru_2_Ph-Croc** during the second oxidation (**left**) with corresponding magnification of the IR/NIR range (**right**). The broken blue and orange lines denote the positions of the individual sub-bands according to spectral deconvolution.

Further oxidation to the dication is characterized by the continued intensity gain of all bands in the visible and near infrared (NIR) regions of the electromagnetic spectrum (Figure 10, orange spectral lines). A closer look at the NIR spectrum (right panel in Figure 10) provides a more detailed account of the subtle changes in the positions and intensities of the individual peaks as obtained from spectral deconvolution (broken blue and orange spectral lines). This close-up view also reveals that no additional IVCT band was present in **^2^Ru_2_Ph-Croc^•+^** which would bleach during the second oxidation. While the smallest feature, formally at 10,770 cm^−1^ (930 nm), remains basically unaltered during the second oxidation ($\tilde{\nu }$_max_ = 10,775 cm^−1^), the second peak, formerly located at 9300 cm^−1^ (1085 nm), is slightly shifted blue to 9370 cm^−1^ (1067 nm) whilst also becoming the most prominent NIR feature. The NIR peak at the lowest energy is shifted blue from 7830 cm^−1^ (1275 nm) to 8065 cm^−1^ (1240 nm). Such blue shift of the NIR absorption bands during successive charging processes of macrocycles built from divinylphenylene diruthenium (Ru_2_Ph) complex fragments was also observed for the carboxylate-bridged variants, in particular when the individual Ru_2_Ph subunits are bridged by relatively short linkers. This holds true even in cases, where the intermediate one-electron oxidized forms could not be observed as se-parate species due to a too close proximity of the individual one-electron waves. These shifts were hence ascribed to decreased electron-donating capabilities of the bis(chelating) bridging ligand as peripheral redox sites are oxidized stepwise, i.e., to inductive effects [86]. The fact that this influence is particularly large in the smaller macrocycle **^2^Ru_2_Ph-Croc** with an even closer transannular distance between the two Ru_2_Ph fragments supports this notion further.

Dioxidized **^2^Ru_2_Ph-Croc^2+^** has again an inherently symmetric electron density distribution as both Ru_2_Ph units are present in their singly oxidized states. This precludes IVCT transitions between identical subunits and leads to again a good match between experimental and calculated spectra if a triplet state is assumed. As it is evident from the computed spectrum shown as Figure S15 in the Supplementary Materials, the NIR absorption is calculated to comprise of two transitions at λ_calc_ = 923 and 928 nm of highly mixed cha-racter, which involve the highest two occupied and lowest two unoccupied spin orbitals β-HOSO−1 to β-LUSO+1. Both members of each pair are again nearly degenerate sets of in- and out-of-phase combinations of MOs confined to the Ru_2_Ph entities, as was the case for the neutral form. However, in reality, individual excitations are confined to only one open-shell Ru_2_Ph**^•+^** entity. Our TD-DFT calculations correctly predict the presence of the charge-transfer transition from the two Ru_2_Ph^•+^ fragments to the croconate linkers. The invariance of the absorption feature at λ_max_ = 410, corresponding to the croconate-based π-π* transition, both in terms of intensity and energy, further indicates a redox-innocent behavior of this ligand within this macrocyclic system.

**Figure 11:**
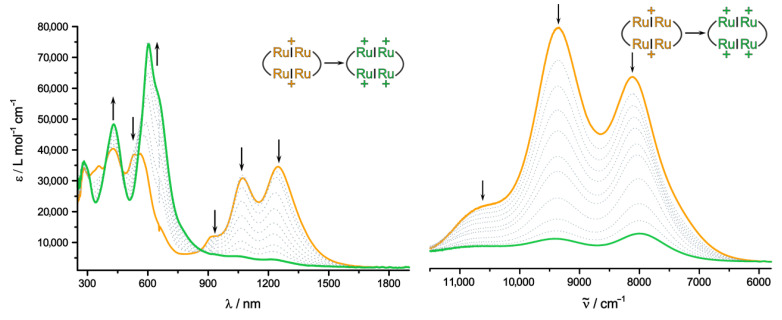
Changes in the UV/Vis/NIR spectrum (DCE, 0.05 M NBu_4_BAr^F^_24_, r.t.) of **^2^Ru_2_Ph-Croc** during the third/fourth oxidations (**left**) with corresponding excerpt from the IR/NIR range (**right**).

As was found in the IR experiment, UV/Vis/NIR spectroelectrochemistry fails to resolve the third and fourth oxidations of **^2^Ru_2_Ph-Croc** into individual one-electron steps. This means that the intermediate trication has no specific absorption profile that would set it apart from mixtures of the two- and fourfold oxidized forms. The evolution of the spectra during these final two oxidations are displayed in Figure 11. The final two-electron oxidation entails the continuous bleach of all NIR bands, signaling the absence of mixed-valent Ru_2_Ph**^•+^** moieties within the macrocycle (note that the small residual intensity in the NIR signals that complete conversion to the tetracation could not be achieved whilst maintaining reasonable voltage levels so as to avoid decomposition). We also wish to point out that at no stage of the final oxidation we observed the initial growth and decrease of a new NIR band that would indicate IVCT between the monooxidized Ru_2_Ph**^•+^** and the dioxidized Ru_2_Ph^2+^ entities (note that, even in the absence of a redox splitting, the trication will be the dominant species at some point during the electrolysis) [27]. Comparison between experimental and calculated UV/Vis/NIR spectra suggests that **^2^Ru_2_Ph-Croc^4+^** seems to behave analogous to the precursor complex and other macrocycles containing dioxidized Ru_2_Ph^2+^ fragments. Thus, the singlet state constitutes the dominant form at r.t.

The UV/Vis spectrum of **^2^Ru_2_Ph-Croc^4+^** is dominated by a strong absorption band at λ_max_ = 605 nm, which is very characteristic to the bipolaron state of dioxidized divinylphenylene diruthenium complexes. The increased ring strain and the ensuing unusually large torsion at the vinyl phenylene linkages in the present macrocycle seem to have a detrimental effect on the absorptivity, as the molar extinction coefficient is smaller by ca. 25% as compared to those obtained for related isophthalate-bridged systems [27,29,32,86]. This band is replicated well by the quantum chemical calculations (λ_calc_ = 574 nm). Corresponding contour and EDDM plots are shown as Figure S16 in the Supplementary Materials. The second relevant absorption feature is the π-π* transition on the croconate bridging ligands. While the energy of this band (λ_max_ = 410 nm) is still unaltered, it gains further in intensity. Our quantum chemical calculations put it at λ_max_ = 400 nm with main contributions from the HOMO and LUMO+2, the latter corresponding to the HOMO−2 and LUMO of the neutral state. The overall scenario that emerges from our investigations is therefore the same as that in the dicarboxylate-bridged macrocyclic structures in that both Ru_2_Ph^n+^ chromophores act independently of each other and “just happen to be chemically linked”.

### 2.4. EPR Spectroscopy

All accessible oxidized forms of **^2^Ru_2_Ph-Croc** were also investigated by means of EPR spectroscopy. Samples of the respective oxidized forms for EPR detection were generated by chemically oxidizing **^2^Ru_2_Ph-Croc** with appropriate amounts of ferrocenium hexa-fluorophosphate (FcH^+^ PF_6_^−^*, E*_1/2_ = 0 mV; 0.8 equiv. for selective formation of **^2^Ru_2_Ph-Croc^+^**, 2.5 equiv. for quantitative formation of **^2^Ru_2_Ph-Croc^2+^**), or 4.4 equiv. of 1,1′-diacetylferrocenium hexafluoroantimonate (Ac_2_Fc^+^ SbF_6_^−^*, E*_1/2_ = 490 mV for **^2^Ru_2_Ph-Croc^4+^**). Their EPR-inactivity above the temperature of liquid helium renders these ferrocenium salts ideally suited for this purpose. The identity of the respective oxidized species was verified by comparing the characteristic Ru(CO) stretches of the chemically oxidized complexes to the spectra obtained via IR-SEC.

**Figure 12:**
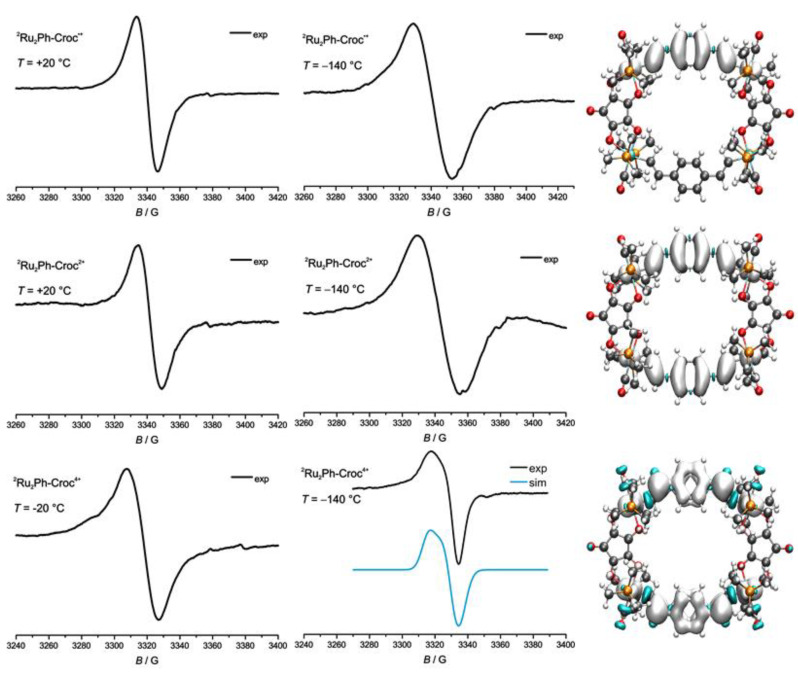
Experimental EPR spectra of **^2^Ru_2_Ph-Croc^+^** (**top left**), **^2^Ru_2_Ph-Croc^2+^** (**middle left**), and **^2^Ru_2_Ph-Croc^4+^** (**bottom left**) at +20 °C in CH_2_Cl_2_ solution or at −140 °C in the frozen glass (**middle panel** with simulated spectrum of **^2^Ru_2_Ph-Croc^4+^** in blue (**bottom middle**). Calculated spin density distribution in **^2^Ru_2_Ph-Croc^+^** (**top right**), **^2^Ru_2_Ph-Croc^2+^** (**middle right**), and **^2^Ru_2_Ph-Croc^4+^** (**bottom right**).

The radical cation **^2^Ru_2_Ph-Croc^•+^** is characterized by an isotropic signal in fluid solution, over a temperature range of +20 °C down to −50 °C, and in the frozen glass at −140 °C (see top left and middle panels of Figure 12). The *g*_iso_-value of 2.012 is notably close to the free electron value *g*_e_ = 2.0023, but larger than that of 1.998 for the tetra*^n^*butylammonium salt of the croconate radical anion (see Figure S17 of the Supporting Materials). The position is typical of the radical cations of divinylarylene-bridged diruthenium complexes and indicates that the spin-bearing orbital is dominated by the π-conjugated divinylphenylene linker, despite the relatively large torsion around the Ru-CH=CH-C_6_H_4_ linkages. Quantum chemical calculations indeed place the entire unpaired spin density at the oxidized Ru_2_Ph**^•+^** site (note, however that the calculations falsely predict equal spin densities on both Ru_2_Ph entities as shown in Figure S18 in the Supplementary Materials). Oxidation of the second Ru_2_Ph entity to furnish the dication **^2^Ru_2_Ph-Croc^2+^** results in a EPR spectrum, which is virtually identical to that of the monocation, in fluid solution and in the frozen glass (see the middle row of Figure 12). This underlines the localized nature of the two mutually independent spins in the dication **^2^Ru_2_Ph-Croc^2+^**. DFT again confirms this view as it assigns no probability density of the unpaired electrons to the croconate bridges.

As it is typical for compounds containing the dioxidized Ru_2_Ph^2+^ complex fragment, the tetracation **^2^Ru_2_Ph-Croc^4+^** is also EPR-active at room temperature, although the electronic spectra were better matched by assuming a singlet ground state. The open-shell form is hence thermally accessible. The relatively high charge on this small cyclic molecule renders **^2^Ru_2_Ph-Croc^4+^** too unstable to allow for EPR measurements at +20 °C without competing decomposition. The spectrum in fluid solution was hence recorded at −20 °C. Under these conditions, a slightly anisotropically broadened signal is observed with a *g*_ave_-value of 2.026 (see Figure 12, bottom left), which is again typical of such species [27,29,32,86]. The slightly increased *g* value points to higher spin densities at the ruthenium ions. This also becomes evident from the axial EPR signal detected at −140 °C with associated *g*-values of *g*_z_ = 2.040 and *g*_x_, *g*_y_ = 2.015. Mirroring the charge distributions, only 1% of the unpaired spin density are computed to reside on the croconate ligands in the open-shell quintet state.

## 3. Conclusions

Three lessons can be learned from our study of the tetraruthenium metallamacro-cyclic complex **^2^Ru_2_Ph-Croc**: (i) Incorporation of an intrinsically redox-active ligand into the coordination sphere of a likewise redox-active metal-coligand entity does not necessarily guarantee that this ligand retains this property in the resulting complex. Despite very similar redox potentials of the croconate (C_5_O_5_^2−^) ligands and the employed {Cl(P*^i^*Pr_3_)_2_(CO)Ru}_2_(µ-CH=CH-C_6_H_4_-CH=CH) (**Ru_2_Ph-Cl**) building blocks, all accessible oxidations of **^2^Ru_2_Ph-Croc** are confined to the Ru_2_Ph entities. (ii) In spite of the sizable redox splittings between the first and, in the very weakly ion pairing NBu_4_[B{C_6_H_3_(3,5-CF_3_)}_4_]^−^ (NBu_4_BAr^F^_24_) electrolyte, also the second charging processes of the two {Ru}_2_(µ-CH=CH-C_6_H_4_-CH=CH) entities, and in spite of the specific IR, UV/Vis profiles of the mo-nocation **^2^Ru_2_Ph-Croc^•+^**, even with detectable Ru(CO) band shifts of all Ru(CO) nodes, the mixed-valent forms exhibit different charge states on the opposite Ru_2_Ph^n+^ entities and are strictly valence-localized species. **^2^Ru_2_Ph-Croc** therefore constitutes a particularly instructive and potentially misleading example of a so-called “pretender” [78], which owes the ensuing thermodynamic stabilities of its mixed-valent forms exclusively to electrostatic interactions. In the case of **^2^Ru_2_Ph-Croc**, this is probably due to the very small dimensions of the macrocycle and the close spatial proximity of the Ru_2_Ph electrophores. (iii) The clear identification of a specific intervalence charge transfer (IVCT) band is about the only truly reliable indicator of electronic coupling in mixed-valent systems and must be critically evaluated. This is particularly important as common computational approaches based on (TD)-DFT are still prone to overestimating π-delocalization effects.

## 4. Experimental Section

### 4.1. Computational Details

The ground state electronic structures of the (model) complexes were calculated by density functional theory (DFT) methods using the *Gaussian 16* program packages [94]. Geometry optimizations were performed without any symmetry constraints. Open-shell systems were calculated by the unrestricted Kohn-Sham approach (UKS). Geometry optimization and subsequent vibrational analysis was performed in solvent media. Solvent effects were described by the polarizable continuum model (PCM) with standard parameters for dichloromethane or 1,2-dichloroethane [95,96]. Explementary input files are included as Tables S4–S6 in the Supplementary Materials. The output values of the vibrational analysis were corrected for their offset in zero-point energies dependent on the utilized combination of functional and basis set functions by multiplication with a vibrational frequency scaling factor. This factor is 0.950 for the combination of PBE0/6-31G(d) (see the NIST Standard Reference Database. Precomputed vibrational scaling factors. https://cccbdb.nist.gov/vibscalejust.asp (accessed on 27 August 2021) Electronic spectra were calculated by the TD-DFT method on optimized geometries. The quasirelativistic Wood-Boring small-core pseudopotentials (MWB) [97,98] and the corresponding optimized set of basis functions for Ru atoms [99] as well as 6-31G(d) polarized double-ζ basis sets for the remaining atoms [100] were employed together with the Perdew, Burke, Ernzerhof exchange and correlation functional (PBE0 aka. PBE1PBE) [101,102] The *GaussSum* program package was used to analyze the results [102], while the visualization of the results was performed with the *Avogadro* program package [103]. Graphical representations of molecular orbitals were generated with *GNU Parallel* [104] and plotted using the *VMD* program package [105] in combination with *POV-Ray* [106]. 

### 4.2. Materials and Methods

^1^H-, ^13^C{^1^H}- and ^31^P{^1^H} spectra were recorded using Bruker Avance III 400 MHz (*B*_H_ = 400 MHz, *B*_P_ = 162 MHz) and *Bruker* Avance NEO 800 (*B*_H_ = 800 MHz, *B*_C_ = 202 MHz) spectrometers obtained from Bruker BioSpin MRI GmbH, Karslruhe, Germany. Spectral shifts are given in ppm and were referenced to the residual protonated solvent (^1^H), the solvent signal (^13^C), or to external for 87% H_3_PO_4_.

All electrochemical experiments were performed with a computer-controlled *BASi* potentiostat from Bioanalytical Systems, West Lafayette, IN, USA, in a custom-built, cylindrical, vacuum-tight one-compartment cell. A spiral-shaped Pt wire and an Ag wire, covered with electrodeposited AgCl, as the counter and pseudo-reference electrodes are sealed into glass capillaries that are introduced via Quickfit screws at opposite sides of the cell. A platinum working electrode (diameter 1.6 mm, from *BASi* is introduced through the top port via a PTFE screw cap with a suitable fitting. It is polished with first 1.0 mm and then 0.25 mm diamond pastes (Buehler-Wirtz, Lake Bluff, IL, USA) before measurements. To exclude the presence of O_2_ and N_2_, the cell and solvent (ca. 7 mL of CH_2_Cl_2_) are purged by bubbling a continuous stream of argon through the solution for several minutes. NBu_4_PF_6_ (0.1 M) or NBu_4_BAr^F^_24_ (0.05 M) were used as the supporting electrolyte. Internal referencing was done by the addition of equimolar amounts of decamethylferrocene (Cp*_2_Fe) after all data of interest had been acquired. Final referencing was done against the ferrocene/ferrocenium (Cp_2_Fe^0/+^) redox couple with *E*_1/2_(Cp*_2_Fe^0/+^) = −550 mV with NBu_4_PF_6_ or *E*_1/2_(Cp*_2_Fe^0/+^) = −620 mV with NBu_4_BAr^F^_24_ as the supporting electrolyte. 

IR and UV/Vis/NIR spectroelectrochemistry was conducted inside a custom-built optically transparent thin-layer electrochemical (OTTLE) according the design of Hartl and coworkers [81]. It comprises of a Pt-mesh working and counter electrode and a thin silver plate as the pseudo-reference electrode sandwiched between the CaF_2_ windows of a conventional liquid IR cell. For the SEC measurements, increased supporting electrolyte concentrations of 0.25 M for NBu_4_PF_6_ or 0.1 M for NBu_4_BAr^F^_24_ and 1,2-dichloroethane as the solvent were used. For the spectroelectrochemical experiments a Wenking POS2 potentiostat (Bank Elektronik-Intelligent Controls GmbH, Pohlheim, Germany) was used. UV/Vis/NIR spectra were obtained on a TIDAS fiberoptic diode array spectrometer (combined MCS UV/NIR and PGS NIR instrumentation) from *J&M* Analytik AG, Essingen, Germany in a range between 250 nm to 2100 nm in *HELLMA* quartz cuvettes with 0.1 cm optical path length. Electron paramagnetic resonance (EPR) studies were performed on a tabletop X-band spectrometer MiniScope 400 with the matching temperature controller model H03, both manufactured by Magnettec GmbH, Berlin, Germany. Samples for EPR spectroscopy were prepared from the diamagnetic forms by chemical oxidation with either ferrocenium hexafluorophosphate (FcHPF_6_*, E*_1/2_ = 0 mV; 0.8 equiv. for selective formation of **^2^Ru_2_Ph-Croc^+^**, 2.5 equiv. for quantitative formation of **^2^Ru_2_Ph-Croc^2+^**), or 4.4 equiv. of 1,1′-diacetylferrocenium hexafluoroantimonate (Ac_2_FcSbF_6_*, E*_1/2_ = 490 mV for **^2^Ru_2_Ph-Croc^4+^**) as the oxidants. The EPR spectrum of **^2^Ru_2_Ph-Croc^4+^** was simulated with *MATLAB* using the *EasySpin* tool box (v. 5.2.8, see: http://easyspin.org/ (accessed on 27 August 2021) [107], using the core-function ‘garlic’ for isotropic signals obtained from measurements in fluid solution and ‘pepper’ for anisotropic signals obtained from measurements in the frozen glass. Mass spectrometric measurements were conducted by Dr. Nicole Orth within the facilities of Prof. Dr. Ivanović-Burmazović at the Friedrich-Alexander-University of Erlangen-Nürnberg on an UHR-ToF Bruker Daltonik maXis plus instrument (Bruker Daltonik GmbH, Bremen, Germany), an ESI-quadrupole time-of-flight (qToF) mass spectrometer capable of resolution of at least 60.000 FWHM, coupled to a Bruker Daltonik cryospray unit. Detection was done in the positive-ion mode with the source voltage set to 4.5 kV. The dry gas (N2) was held at −85 °C and the spray gas at −90 °C. The spectrometer was calibrated with ESI-ToF Low Concentration Tuning Mix from Agilent prior to every measurement. All measurements were carried out using dichloromethane as the solvent. All measurements were carried out using dichloromethane as the solvent. FT-IR spectra were recorded using a Bruker Tensor II FT-IR spectrometer (Bruker, Billerica, MA, USA).

X-Ray diffraction analysis was performed on a STOE IPDS-II diffractometer (STOE&CIE GmbH, Darmstadt, Germany) equipped with a graphite monochromated MoK_α_ radiation source (λ = 0.71073 Å) and an image plate detection system at *T* = 100.15 K. Using *Olex2* [71], the structures were solved with the *ShelXT* [108] structure solution program using Intrinsic Phasing and refined with the *ShelXL* [108] refinement package using Least Squares minimization. Hydrogen atoms were introduced at their calculated positions. Structure plots were generated with the *Platon* program [70].

### 4.3. Synthesis

Dipotassium croconate monohydrate [69] and bis(tetra-*n*-butylammonium) croconate tetrahydrate (**NBu_4_Croc**) [80] as well as the complex {Ru(CO)Cl(P*^i^*Pr_3_)_2_}_2_(µ-1,4-CH=CH-C_6_H_4_-CH=CH) (**Ru_2_Ph-Cl**) [47] were prepared by published procedures.

#### 4.3.1. Synthesis and Isolation of Pure **^2^Ru_2_Ph-Croc**

The procedure detailed below has been optimized to enable the isolation of the pure tetranuclear macrocycle. Deviation from this combination of solvents as well as concentrations, reaction temperature and time is discouraged and will very probably lead to impure material. 180 mg (0.16 mmol, 1 eq) of **Ru_2_Ph-Cl** were dissolved in 6 mL of benzene and 39 mg (0.17 mmol, 1.06 eq) of potassium croconate monohydrate were dissolved in 3 mL of H_2_O. The combined emulsion was stirred for 19 hours at 35 °C. The precipitated solid was separated from the emulsion by centrifugation. The greenish brown solid was dried under vacuo. 49 mg (0.021mmol) of brown **^2^Ru_2_Ph-Croc** were obtained, corresponding to a yield of 21%.

^1^H-NMR (800 MHz, CD_2_Cl_2_, 300 K): δ[ppm] 9.29 (d, ^3^*J*_HH_ = 15.7 Hz, 4H, H_α-vinyl_), 7.15 (s, 8H, H_phenylene_), 6.21 (d, ^3^*J*_HH_ = 15.7 Hz, 4H, H_β-vinyl_), 2.34–2.26 (m, 24H, PC*H*CH_3_), 1.19–1.13 (m, 144 H, PCHC*H*_3_).

^13^C{^1^H}-NMR (202 MHz, CD_2_Cl_2_, 300 K): δ[ppm] = 208.1 (t, ^3^*J*_CP_ = 13.0 Hz, *C*O), 199.1/184.8/180.6 (s, C_croconate_), 155.5 (t, ^3^*J*_CP_ = 13.0 Hz, C_α-vinyl_), 137.5 (s, C_phenylene_), 134.1 (s, C_β-vinyl_), 124.4 (s, C_phenylene_), 25.2 (vt, ^2^*J*_CP_ = 9.0 Hz, P*C*HCH_3_), 20.5 (s, PCH*C*H_3_) 19.8 (s, PCH*C*H_3_).

^31^P{^1^H}-NMR (162 MHz, CD_2_Cl_2_, 300 K): δ[ppm] 35.10 (s, *P*CHCH_3_).

MS (ESI[+], CH_2_Cl_2_): calcd for ^2^Ru_2_Ph-Croc^+^ = 2336.77, found 2336.78; calcd for ^2^Ru_2_Ph-Croc^2+^ = 1167.39, found 1167.39.

#### 4.3.2. Isolation of Mixtures of **^2^Ru_2_Ph-Croc** and **^4^Ru_2_Ph-Croc**

The supernatant obtained after centrifugation of the reaction mixture from Section 4.3.1 was diluted with dichloromethane and water. The layers were separated and the organic layer washed twice with 5 mL of water to remove excess croconate ligand. The organic layer was subsequently dried over Na_2_SO_4_ (the use of MgSO_4_ is discouraged due to the instability of vinyl ruthenium species towards Lewis acids). After solvent removal under reduced pressure the brown residue was washed with small portions of first MeOH and the hexane (<1 mL) utilizing ultra-sonication and centrifugation to remove ruthenium-containing decomposition products. The obtained reddish brown solid contains both, ***^2^Ru_2_Ph-Croc*** *and **^4^Ru_2_Ph-Croc***, which were found to be impossible to separate and purify to a satisfying degree.

Assignment of the ^1^H and ^31^P{^1^H} resonances for ***^4^Ru_2_Ph-Croc*** from spectra containing both kinds of macrocycles was performed based on the results for pure ***^2^Ru_2_Ph-Croc***.

^1^H-NMR (400 MHz, CD_2_Cl_2_, 300 K): δ[ppm] 8.88 (d, ^3^*J*_HH_ = 16.4 Hz, 8H, H_α-vinyl_), 7.05 (s, 16 H, H_phenylene_), 6.30 (d, ^3^*J*_HH_ = 15.7 Hz, 8H, H_β-vinyl_), 2.39–2.16 (m, PC*H*CH_3_), 1.43–1.13 (m, PCHC*H*_3_).

^31^P{^1^H}-NMR (162 MHz, CD_2_Cl_2_, 300 K): δ[ppm] 35.31 (s, *P*CHCH_3_).

MS (ESI[+], CH_2_Cl_2_): calcd for ^4^Ru_2_Ph-Croc^2+^ = 2336.77, found 2336.78; calcd for ^4^Ru_2_Ph-Croc^3+^ = 1556.51, found 1556.52.

## Figures and Tables

**Table 1:** Electrochemical data of **^2^Ru_2_Ph-Croc** in CH_2_Cl_2_ in either 0.1 M NBu_4_PF_6_ or NBu_4_BAr^F^_24_ as the supporting electrolyte as well as comparative data for **Ru_2_Ph-Cl** and ^2^**Ru_2_Ph-*^m^*Py**.

	Electrolyte	*E*_1/2_^0/+^ [mV]	*E*_1/2_^+/2+^ [mV]	Δ*E*_1/2_^0/+//+/2+^ [mV]	*E*_1/2_^2+/3+^ [mV]	*E*_1/2_^3+/4+^ [mV]	Δ*E*_1/2_^2+/3+//3+/4+^ [mV]
**^2^** **Ru_2_Ph-Croc**	NBu_4_PF_6_	−134	−17	117	207		0
	NBu_4_BAr^F^_24_	−226	−85	141	245	410	165
**Ru_2_Ph-Cl ^a^**	NBu_4_PF_6_	−75	175	250	-	-	-
	NBu_4_BAr^F^_24_	−180	130	310	-	-	-
**^2^** **Ru_2_Ph-*^m^*Py ^a^**	NBu_4_BAr^F^_24_	−431	−334	97	−39	42	81

^a^ Data for **Ru_2_Ph-Cl** and **Ru_2_Ph-*^m^*Py^a^** from Ref. [27].

**Table 2:** Calculated and experimentally obtained energies of characteristic IR bands of **^2^Ru_2_Ph-Croc** in all accessible oxidation states.

$\tilde{\nu }$ [cm^−1^]							
^2^Ru_2_Ph-Croc	IR Label	Neutral	Monocation	Dication (Singlet)	Dication (Triplet)	Tetracation (Singlet)	Tetracation (Quintet)
**Calculated ^a,b^**	Ru(CO)	1913, 1914	1918, 1920, 1934, 1942	1939, 1942, 1942, 1949	1939, 1939, 1945, 1948	1977, 1978, 1981, 1983	1983, 1984, 1988, 1989
	C=O_Croc_	1652	1656	1655	1659	1664	1661
	C-C_rocking_	1485	1482	1486	1479	1477	1473
**Experimental**	Ru(CO)	1913	1917, 1935, 1949	1941, 1952		1982	
	C=O_Croc_	1630	1636	1639		1644	
	C-C_rocking_	1503	1499	1498		1493	

^a^ Calculated with the pbe1pbe/6-31G(d) functional. ^b^ The computed ground state is indicated in bold writing.

**Table 3:** UV/Vis/NIR data of the complexes in their various oxidation states ^a^.

	*λ*_max_ [nm] (ε [M^−1^ cm^−1^])	TD-DFT calc. Transitions ^b^		
		*λ*_calc_ [nm]	Contribution (Minor)	Character
0	550 (8900), 410 (41,100), 350 (73,400)	626–608 431 329	H → L, (H-1 → L+1) H-2 → L H → L+4	ML-L′CT π-π*_Croc_ π-π*_Ru2Ph_
1+	1275 (23,000), 1085 (14,300), 930 (7300), 570 (23,700), 535 (22,900), 410 (41,100), 350 (53,000)	2171 1011 663 505	βH → βL βH-1 → βL βH → βL+2 αH-1 → αL	IVCT (n.o.) ML-L′CT ML-L′CT ML-L′CT
2+ ^b^	1240 (34,600), 1067 (30,800), 930 (11,900), 570 (38,800), 535 (38,800), 410 (41,100), 350 (34,600)	928, 923 529	βH → βL, (βH-1 → βL+1) αH-1 → αL	CR ML-L′CT
4+ ^c^	650 (sh), 605 (7,6500), 410 (48,400)	940–923 574 400	H → L, (H-1 → L+1) H-2 → L, (H-3 → L+1) H → L+2	L′-MLCT (n.o.) π-π*_Ru2Ph_ π-π*_Croc_

^a^ in DCE/0.05 M NBu_4_BAr^F^_24_; ^b^ for the triplet state; ^c^ for the singlet state.

## Data Availability

All relevant data of this study are in the main document or in the Supplementary Materials.

## Supplementary Materials

The following are available online: Figures S1 and S2: NMR spectra of mixtures of macrocycles **^2^Ru_2_Ph-Croc** and **^4^Ru_2_Ph-Croc**; Figure S3–S5: NMR spectra of pure **^2^Ru_2_Ph-Croc**; Figures S6 and S7: mass spectra of the base peak of the di-and the tricationics of pure macrocycle **^2^Ru_2_Ph-Croc**; Tables S1–S3: Crystal and refinement data (Table S1), bond lengths (Table S2) and bond angles (Table S3) for the crystallographic structure of macrocycle **^2^Ru_2_Ph-Croc**; Figure S8: Plot of the crystallographically unique unit of the structure of **^2^Ru_2_Ph-Croc** with atom numbering; Figure S9: cyclic voltammograms showing the reductive scan of **^2^Ru_2_Ph-Croc** in two different eletcrolytes; Figure S10: Anodic scan of the tetrabutylammonium salt of croconate in 0.1 M NBu_4_PF_6_/CH_2_Cl_2_; Figure S11: spectroscopic changes in the IR upon oxidation of (NBu_4_)_2_(croconate); Figure S12: IR spectra of **^2^Ru_2_Ph-Croc^n+^** (n = 0, 1, 2, 4) in CH_2_Cl_2_; Figures S13–S16: TD-DFT computed absorption spectra of **^2^Ru_2_Ph-Croc^n+^** (n = 0, 1, 2, 4) along with graphical representations of the involved MOs and the differences in electron density distributions corresponding to the main excitations; Figure S17: EPR spectrum of the croconate radical anion; Figure S18: calculated spin densities of **^2^Ru_2_Ph-Croc^+^**, **^2^Ru_2_Ph-Croc^2^**^+^ (triplet state) and **^2^****Ru_2_Ph-Croc^4+^**. The crystallographic data for **^2^Ru_2_Ph-Croc** were deposited at the Cambridge Crystallographic Data Centre under the deposition number CCDC 2094893. The data can be obtained free of charge via www.ccdc.cam.ac.uk/data_request/cif, or by emailing data_request@ccdc.cam.ac.uk, or by contacting The Cambridge Crystallographic Data Centre, 12 Union Road, Cambridge CB2 1EZ, UK; fax: +44-1223-336033.

## Sample Availability

Not available.

## Abbreviations

The following abbreviations are used in this manuscript:

[9]aneS_3_	1,4,7-trithiacyclononane
BAr^F^_24_	[B{C_6_H_3_(2,5-CF_3_)}]^−^
CT	charge transfer
EPR	electron paramagnetic resonance
FcH/FcH^+^	ferrocene/ferrocenium
FMO	frontier molecular orbital
HOMO	energetically highest occupied molecular orbital
IR	infrared
IVCT	intervalence charge transfer
LUMO	energetically lowest unoccupied molecular orbital
LMCT	ligand-to-metal charge transfer
MLCT	metal-to-ligand charge transfer
NBO	natural bond order
NBu_4_	tetra*^n^*butylammonium
NIR	near infrared
NMR	nuclear magnetic resonance
Ru_2_Ph	{Ru(CO)(P^i^Pr_3_)_2_}(1,4-CH=CH-C_6_H_4_-CH=CH)
UV/Vis/NIR	electron spectroscopy in the ultraviolet, visible and near infrared
TD-DFT	time-dependent density functional theory

## References

[1] Khoshbin M.S., Ovchinnikov M.V., Mirkin C.A., Zakharov L.N., Rheingold A.L. (2005). Binuclear Ruthenium Macrocycles Formed via the Weak-Link Approach. Inorg. Chem..

[2] Chakrabarty R., Mukherjee P.S., Stang P.J. (2011). Supramolecular Coordination: Self-Assembly of Finite Two- and Three-Dimensional Ensembles. Chem. Rev..

[3] Datta S., Saha M.L., Stang P.J. (2018). Hierarchical Assemblies of Supramolecular Coordination Complexes. Acc. Chem. Res..

[4] Han Y.-F., Jin G.-X. (2014). Half-Sandwich Iridium- and Rhodium-based Organometallic Architectures: Rational Design, Synthesis, Characterization, and Applications. Acc. Chem. Res..

[5] Cook T.R., Stang P.J. (2015). Recent Developments in the Preparation and Chemistry of Metallacycles and Metallacages via Coordination. Chem. Rev..

[6] Zhang Y.-Y., Gao W.-X., Lin L., Jin G.-X. (2017). Recent advances in the construction and applications of heterometallic macrocycles and cages. Coord. Chem. Rev..

[7] Lu Y., Zhang H.-N., Jin G.-X. (2018). Molecular Borromean Rings Based on Half-Sandwich Organometallic Rectangles. Acc. Chem. Res..

[8] Croue V., Goeb S., Szaloki G., Allain M., Salle M. (2016). Reversible Guest Uptake/Release by Redox-Controlled Assembly/Disassembly of a Coordination Cage. Angew. Chem. Int. Ed..

[9] Szalóki G., Croué V., Carré V., Aubriet F., Alévêque O., Levillain E., Allain M., Aragó J., Ortí E., Goeb S. (2017). Controlling the Host–Guest Interaction Mode through a Redox Stimulus. Angew. Chem. Int. Ed..

[10] Goeb S., Sallé M. (2021). Electron-rich Coordination Receptors Based on Tetrathiafulvalene Derivatives: Controlling the Host–Guest Binding. Acc. Chem. Res..

[11] Schulze M., Kunz V., Frischmann P.D., Würthner F. (2016). A supramolecular ruthenium macrocycle with high catalytic activity for water oxidation that mechanistically mimics photosystem II. Nat. Chem..

[12] Kunz V., Schulze M., Schmidt D., Würthner F. (2017). Trinuclear Ruthenium Macrocycles: Toward Supramolecular Water Oxidation Catalysis in Pure Water. ACS Energy Lett..

[13] Kunz V., Lindner J.O., Schulze M., Roehr M.I.S., Schmidt D., Mitric R., Würthner F. (2017). Cooperative water oxidation catalysis in a series of trinuclear metallosupramolecular ruthenium macrocycles. Energy Environ. Sci..

[14] Schindler D., Meza-Chincha A.-L., Roth M., Würthner F. (2021). Structure-Activity Relationship for Di- up to Tetranuclear Macrocyclic Ruthenium Catalysts in Homogeneous Water Oxidation. Chem.-Eur. J..

[15] Dinolfo P.H., Hupp J.T. (2004). Tetra-Rhenium Molecular Rectangles as Organizational Motifs for the Investigation of Ligand-Centered Mixed-Valency: Three Examples of Full Delocalization. J. Am. Chem. Soc..

[16] Dinolfo P.H., Williams M.E., Stern C.L., Hupp J.T. (2004). Rhenium-Based Molecular Rectangles as Frameworks for Ligand-Centered Mixed-Valency and Optical Electron Transfer. J. Am. Chem. Soc..

[17] Dinolfo P.H., Lee S.J., Coropceanu V., Brédas J.-L., Hupp J.T. (2005). Borderline Class II/III Ligand-Centered Mixed Valency in a Porphyrinic Molecular Rectangle. Inorg. Chem..

[18] Dinolfo P.H., Coropceanu V., Brédas J.-L., Hupp J.T. (2006). A New Class of Mixed-Valence Systems with Orbitally Degenerate Organic Redox Centers. Examples Based on Hexa-Rhenium Molecular Prisms. J. Am. Chem. Soc..

[19] Piotrowski H., Polborn K., Hilt G., Severin K. (2001). A self-Assembled Metallamacrocyclic Ionophore with High Affinity and Selectivity for Li^+^ and Na^+^. J. Am. Chem. Soc..

[20] Shan N., Vickers S.J., Adams H., Ward M.D., Thomas J.A. (2004). Switchable Electron-Transfer Processes in a Mixed-Valence, Kinetically Locked, Trinuclear Ru^II^ Metallamacrocycle. Angew. Chem. Int. Ed. Eng..

[21] Shan N., Ingram J.D., Easun T.L., Vickers S.J., Adams H., Ward M.D., Thomas J.A. (2006). Kinetically locked, trinuclear Ru^II^ metallo-macrocycles—synthesis, electrochemical, and optical properties. Dalton Trans..

[22] Maurer J., Linseis M., Sarkar B., Schwederski B., Niemeyer M., Kaim W., Záliš S., Anson C., Zabel M., Winter R.F. (2008). Ruthenium Complexes with Vinyl, Styryl, and Vinylpyrenyl Ligands: A Case of Non-Innocence in Organometallic Chemistry. J. Am. Chem. Soc..

[23] Wuttke E., Hervault Y.-M., Polit W., Linseis M., Erler P., Rigaut S., Winter R.F. (2014). Divinylphenylene- and Ethynylvinylphenylene-Bridged Mono-, Di-, and Triruthenium Complexes for Covalent Binding to Gold Electrodes. Organometallics.

[24] Abdel-Rahman O.S., Maurer J., Záliš S., Winter R.F. (2015). Ruthenium Styryl Complexes with Ligands Derived from 2-Hydroxy- and 2-Mercaptopyridine and 2-Hydroxy- and 2-Mercaptoquinoline. Organometallics.

[25] Fink D., Linseis M., Winter R.F. (2018). Constitutional Isomers of Macrocyclic Tetraruthenium Complexes with Vastly Different Spectroscopic and Electrochemical Properties. Organometallics.

[26] Fink D., Bodensteiner M., Linseis M., Winter R.F. (2018). Macrocyclic Triruthenium Complexes Having Electronically Coupled Mixed-Valent States. Chem. Eur. J..

[27] Scheerer S., Linseis M., Wuttke E., Weickert S., Drescher M., Tröppner O., Ivanović-Burmazović I., Irmler A., Pauly F., Winter R.F. (2016). Redox-Active Tetraruthenium Macrocycles Built from 1,4-Divinylphenylene-Bridged Diruthenium Complexes. Chem. Eur. J..

[28] Fink D., Weibert B., Winter R.F. (2016). Redox-active tetraruthenium metallacycles: Reversible release of up to eight electrons resulting in strong electrochromism. Chem. Commun..

[29] Anders P., Rapp M., Linseis M., Winter R. (2018). Tetraruthenium Metallamacrocycles with Potentially Coordinating Appended Functionalities. Inorganics.

[30] Fink D., Orth N., Linseis M., Ivanović-Burmazović I., Winter R.F. (2020). Ring size matters: Supramolecular isomerism in self-assembled redox-active tetra- and hexaruthenium macrocycles. Chem. Commun..

[31] Fink D., Orth N., Linseis M., Ivanović-Burmazović I., Winter R.F. (2020). Structural Versatility and Supramolecular Isomerism in Redox-Active Tetra- and Hexaruthenium Macrocycles. Eur. J. Inorg. Chem..

[32] Fink D., Staiger A., Orth N., Linseis M., Ivanović-Burmazović I., Winter R.F. (2020). Redox-Induced Hydrogen Bond Reorientation Mimicking Electronic Coupling in Mixed-Valent Diruthenium and Macrocyclic Tetraruthenium Complexes. Inorg. Chem..

[33] Kaim W. (2011). Manifestations of Noninnocent Ligand Behavior. Inorg. Chem..

[34] Kaim W. (2012). The Shrinking World of Innocent Ligands: Conventional and Non-Conventional Redox-Active Ligands. Eur. J. Inorg. Chem..

[35] Kaim W. (2016). Electron Transfer Reactivity of Organometallic Compounds Involving Radical-Forming Noninnocent Ligands. Proc. Nat. Acad. Sci. Ind. A.

[36] Ghumaan S., Mukherjee S., Kar S., Roy D., Mobin S.M., Sunoj R.B., Lahiri G.K. (2006). An Experimental and Density Functional Theory Approach Towards the Establishment of Preferential Metal- or Ligand-Based Electron-Transfer Processes in Large Quinonoid-Bridged Diruthenium Complexes [{(aap)2Ru}2(μ-BL2–)]n+ (aap = 2-Arylazopyridine). Eur. J. Inorg. Chem..

[37] Ghumaan S., Sarkar B., Maji S., Puranik V.G., Fiedler J., Urbanos F.A., Jimenez-Aparicio R., Kaim W., Lahiri G.K. (2008). Valence-State Analysis through Spectroelectrochemistry in a Series of Quinonoid-Bridged Diruthenium Complexes [(acac)_2_Ru(μ-L)Ru(acac)_2_]^n^ (n=+2, +1, 0, −1, −2). Chem. Eur. J..

[38] Maji S., Sarkar B., Mobin S.M., Fiedler J., Urbanos F.A., Jimenez-Aparicio R., Kaim W., Lahiri G.K. (2008). Valence-State Alternatives in Diastereoisomeric Complexes [(acac)_2_Ru(μ-QL)Ru(acac)_2_]n (QL^2−^ = 1,4-Dioxido-9,10-anthraquinone,n = +2, +1, 0, −1, −2). Inorganic Chemistry.

[39] Mandal A., Agarwala H., Ray R., Plebst S., Mobin S.M., Priego J.L., Jiménez-Aparicio R., Kaim W., Lahiri G.K. (2014). Sensitivity of the Valence Structure in Diruthenium Complexes as a Function of Terminal and Bridging Ligands. Inorg. Chem..

[40] Ansari M.A., Mandal A., Paretzki A., Beyer K., Kaim W., Lahiri G.K. (2016). Isomeric Diruthenium Complexes of a Heterocyclic and Quinonoid Bridging Ligand: Valence and Spin Alternatives for the Metal/Ligand/Metal Arrangement. Inorg. Chem..

[41] Kaim W., Lahiri G.K. (2019). The coordination potential of indigo, anthraquinone and related redox-active dyes. Coord. Chem. Rev..

[42] Gmelin L. (1825). Über einige merkwürdige, bei der Darstellung des Kaliums nach der Brunner’schen Methode erhaltene Substanzen. Poggendorfs Ann. Phys..

[43] Braga D., Maini L., Grepioni F. (2002). Croconic Acid and Alkali Metal Croconate Salts: Some New Insights into an Old Story. Chem. Eur. J..

[44] Ranganathan A., Kulkarni G.U. (2002). An Experimental Electron Density Investigation of Squarate and Croconate Dianions. J. Phys. Chem. A.

[45] Georgopoulos S.L., Garcia H.C., Edwards H.G.M., Cappa de Oliveira L.F. (2016). Spectroscopic and structural investigation of oxocarbon salts with tetraalkylammonium ions. J. Mol. Struct..

[46] Schleyer P.v.R., Najafian K., Kiran B., Jiao H. (2000). Are Oxocarbon Dianions Aromatic?. J. Org. Chem..

[47] Maurer J., Sarkar B., Schwederski B., Kaim W., Winter R.F., Záliš S. (2006). Divinylphenylene bridged diruthenium complexes bearing Ru(CO)Cl(P*^i^*Pr_3_)_2_ entities. Organometallics.

[48] Linseis M., Winter R.F., Sarkar B., Kaim W., Záliš S. (2008). Multistep Electrochromic Behavior from an Organometallic Tetranuclear Complex of a Tetradonor-Substituted Olefin. Organometallics.

[49] Linseis M., Záliš S., Zabel M., Winter R.F. (2012). Ruthenium Stilbenyl and Diruthenium Distyrylethene Complexes: Aspects of Electron Delocalization and Electrocatalyzed Isomerization of the *Z*-Isomer. J. Am. Chem. Soc..

[50] Pfaff U., Hildebrandt A., Korb M., Oßwald S., Linseis M., Schreiter K., Spange S., Winter R.F., Lang H. (2016). Electronically Strongly Coupled Divinylheterocyclic-Bridged Diruthenium Complexes. Chem. Eur. J..

[51] Wuttke E., Fink D., Anders P., Maria Hoyt A.-L., Polit W., Linseis M., Winter R.F. (2016). Homo- and heterobimetallic 1,4-divinylphenylene- and naphthalene-1,8-divinyl-bridged diruthenium, diosmium and ruthenium osmium complexes. J. Organomet. Chem..

[52] Rotthowe N., Zwicker J., Winter R.F. (2019). Influence of Quinoidal Distortion on the Electronic Properties of Oxidized Divinylarylene-Bridged Diruthenium Complexes. Organometallics.

[53] Doane L.M., Fatiadi A.J. (1982). Electrochemical oxidation of several oxocarbon salts in *N,N*-dimethylformamide. J. Electroanal. Chem. Interfacial Electrochem..

[54] Fabre P.-L., Dumestre F., Soula B., Galibert A.-M. (2000). Spectroelectrochemical behavior in dimethylformamide of pseudo-oxocarbons dianions derived from the croconate dianion. Electrochim. Acta.

[55] Dumestre F., Soula B., Galibert A.-M., Fabre P.-L., Bernardinelli G., Donnadieu B., Castan P. (1998). Synthesis and characterization of cobalt(II) complexes of croconate and dicyanomethylene-substituted derivatives. J. Chem. Soc. Dalton Trans..

[56] Massoud S.S., Vicente R., Fontenot P.R., Gallo A.A., Mikuriya M., Albering J.H., Mautner F.A. (2012). Polynuclear croconato-bridged-copper(II) complexes derived from tri- and tetra-dentate amines. Polyhedron.

[57] Carranza J., Sletten J., Lloret F., Julve M. (2009). Manganese(II) complexes with croconate and 2-(2-pyridyl)imidazole ligands: Syntheses, X-ray structures and magnetic properties. Inorg. Chim. Acta.

[58] Brouca-Cabarrecq C., Trombe J.-C. (1992). f Element croconates 1. Lanthanide croconates—Synthesis, crystal structure and thermal behavior. Inorg. Chim. Acta.

[59] Castro I., Calatayud M.L., Lloret F., Sletten J., Julve M. (2002). Syntheses, crystal structures and magnetic properties of di- and trinuclear croconato-bridged copper(ii) complexes. J. Chem. Soc. Dalton Trans..

[60] Carranza J., Brennan C., Sletten J., Vangdal B., Rillema P., Lloret F., Julve M. (2003). Syntheses, crystal structures and magnetic properties of new oxalato-, croconato- and squarato-containing copper(ii) complexes. New J. Chem..

[61] Wang C.-C., Ke M.-J., Tsai C.-H., Chen I.H., Lin S.-I., Lin T.-Y., Wu L.-M., Lee G.-H., Sheu H.-S., Fedorov V.E. (2009). [M(C_5_O_5_)_2_(H_2_O)_n_]^2−^ as a Building Block for Hetero- and Homo-bimetallic Coordination Polymers: From 1D Chains to 3D Supramolecular Architectures. Cryst. Growth Des..

[62] Mautner F.A., Albering J.H., Vicente R., Louka F.R., Gallo A.A., Massoud S.S. (2011). Copper(II) complexes derived from tripodal tris[(2-ethyl-(1-pyrazolyl)]amine. Inorg. Chim. Acta.

[63] Zheng Y.-R., Stang P.J. (2009). Direct and Quantitative Characterization of Dynamic Ligand Exchange between Coordination-Driven Self-Assembled Supramolecular Polygons. J. Am. Chem. Soc..

[64] Li J.-R., Zhou H.-C. (2010). Bridging-ligand-substitution strategy for the preparation of metal–organic polyhedra. Nat. Chem..

[65] Neogi S., Lorenz Y., Engeser M., Samanta D., Schmittel M. (2013). Heteroleptic Metallosupramolecular Racks, Rectangles, and Trigonal Prisms: Stoichiometry-Controlled Reversible Interconversion. Inorg. Chem..

[66] Garci A., Gupta G., Dalvit C., Therrien B. (2014). Investigating the Formation Mechanism of Arene Ruthenium Metallacycles by NMR Spectroscopy. Eur. J. Inorg. Chem..

[67] Garci A., Marti S., Schurch S., Therrien B. (2014). Insight into the dynamic ligand exchange process involved in bipyridyl linked arene ruthenium metalla-rectangles. RSC Adv..

[68] Burgstahler A.W., Barkhurst R.C. (1968). Preparation of Leuconic Acid from Inositol. Trans. Kansas Acad. Sci..

[69] Williams R.F.X. (1976). Transition Metal Complexes with Organo-Chalcogen Ligands. I. Synthesis of the Dithoiocroconate Dianion. Phosphorus Sulf. Rel. Elem..

[70] Spek A. (2015). PLATON SQUEEZE: A tool for the calculation of the disordered solvent contribution to the calculated structure factors. Acta Cryst. C Cryst. Struct. Commun..

[71] Dolomanov O.V., Bourhis L.J., Gildea R.J., Howard J.A.K., Puschmann H. (2009). OLEX2: A complete structure solution, refinement and analysis program. J. Appl. Cryst..

[72] Dunitz J.D., Seiler P., Czechtizky W. (2001). Crystal Structure of Potassium Croconate Dihydrate, after 175 Years. Angew. Chem. Int. Ed..

[73] Richardson D.E., Taube H. (1984). Mixed-valence molecules: Electronic delocalization and stabilization. Coord. Chem. Rev..

[74] Crutchley R.J., Sykes A.G. (1994). Intervalence Charge Transfer and Electron Exchange Studies of Dinuclear Ruthenium Complexes. Advances in Inorganic Chemistry.

[75] Evans C.E.B., Naklicki M.L., Rezvani A.R., White C.A., Kondratiev V.V., Crutchley R.J. (1998). An Investigation of Superexchange in Dinuclear Mixed-Valence Ruthenium Complexes. J. Am. Chem. Soc..

[76] D’Alessandro D.M., Keene F.R. (2004). A cautionary warning on the use of electrochemical measurements to calculate comproportionation constants for-mixed-valence compounds. Dalton Trans..

[77] Fellows E.A., Keene F.R. (2007). Influence of Anions on Intervalence Charge Transfer (IVCT) in Mixed-Valence Dinuclear Complexes. J. Phys. Chem. B.

[78] Winter R.F. (2014). Half-Wave Potential Splittings Δ*E*_1/2_ as a Measure of Electronic Coupling in Mixed-Valent Systems: Triumphs and Defeats. Organometallics.

[79] Hildebrandt A., Miesel D., Lang H. (2018). Electrostatic interactions within mixed-valent compounds. Coord. Chem. Rev..

[80] Ribeiro M.C., de Oliveira L.F., Gonçalves N.S. (2001). Boson peak in the room-temperature molten salt tetra(n-butyl)ammonium croconate. Phys. Rev. B.

[81] Krejcik M., Danek M., Hartl F. (1991). Simple construction of an infrared optically transparent thin-layer cell: Applications to the redox reactions of ferrocene, Mn_2_(CO)_10_ and Mn(CO)_3_(3,5-di-t-butyl-catecholate)^−^. J. Electroanal. Chem..

[82] Hassenrück C., Mücke P., Scheck J., Demeshko S., Winter R.F. (2017). Oxidized Styrylruthenium–Ferrocene Conjugates: From Valence Localization to Valence Tautomerism. Eur. J. Inorg. Chem..

[83] Záliš S., Winter R.F., Kaim W. (2010). Quantum chemical interpretation of redox properties of ruthenium complexes with vinyl and TCNX type non-innocent ligands. Coord. Chem. Rev..

[84] Scheerer S., Rotthowe N., Abdel-Rahman O.S., He X., Rigaut S., Kvapilová H., Záliš S., Winter R.F. (2015). Vinyl Ruthenium-Modified Biphenyl and 2,2′-Bipyridines. Inorg. Chem..

[85] Chen J., Winter R.F. (2012). Studies on a Vinyl Ruthenium-Modified Squaraine Dye: Multiple Vis/NIR Absorbance Switching through Dye and Substituent-Based Redox Processes. Chem. Eur. J..

[86] Fink D., Orth N., Ebel V., Gogesch F.S., Staiger A., Linseis M., Ivanović-Burmazović I., Winter R.F. (2020). Self-Assembled Redox-Active Tetraruthenium Macrocycles with Large Intracyclic Cavities. Organometallics.

[87] Petrov A.I., Lutoshkin M.A. (2021). TD-DFT assessment of UV-vis spectra palladium and platinum complexes with thiols and disulfides. J. Mol. Model..

[88] Gąsiorski P., Matusiewicz M., Gondek E., Uchacz T., Danel A., Wojtasik K., Vlokh R., Kityk A.V. (2017). Synthesis, UV–Vis spectroscopy and DFT/TDDFT calculations on 6-substituted halogen derivatives of 1,3-diphenyl-1H-pyrazolo [3,4−b] quinoxalines dyes. J. Lumin..

[89] Amat A., Miliani C., Romani A., Fantacci S. (2015). DFT/TDDFT investigation on the UV-vis absorption and fluorescence properties of alizarin dye. Phys. Chem. Chem. Phys..

[90] Kaupp M., Gückel S., Renz M., Klawohn S., Theilacker K., Parthey M., Lambert C. (2016). Electron transfer pathways in mixed-valence paracyclophane-bridged bis-triarylamine radical cations. J. Comput. Chem..

[91] Sakamaki D., Ito A., Tsutsui Y., Seki S. (2017). Tetraaza[14]- and Octaaza[18]paracyclophane: Synthesis and Characterization of Their Neutral and Cationic States. J. Org. Chem..

[92] Kaupp M., Renz M., Parthey M., Stolte M., Würthner F., Lambert C. (2011). Computational and spectroscopic studies of organic mixed-valence compounds: Where is the charge?. Phys. Chem. Chem. Phys..

[93] Parthey M., Kaupp M. (2014). Quantum-chemical insights into mixed-valence systems: Within and beyond the Robin-Day scheme. Chem. Soc. Rev..

[94] Frisch M.J., Trucks G.W., Schlegel H.B., Scuseria G.E., Robb M.A., Cheeseman J.R., Scalmani G., Barone V., Petersson G.A., Nakatsuji H. (2016). Gaussian, Inc., Wallingford CT, Gaussian 09, Revision D.01. https://gaussian.com/.

[95] Mennucci B., Tomasi J. (1997). Continuum solvation models: A new approach to the problem of solute’s charge distribution and cavity boundaries. J. Chem. Phys..

[96] Cossi M., Rega N., Scalmani G., Barone V. (2003). Energies, structures, and electronic properties of molecules in solution with the C-PCM solvation model. J. Comput. Chem..

[97] Dolg M., Wedig U., Stoll H., Preuss H. (1987). Energy-adjusted ab initio pseudopotentials for the first row transition elements. J. Chem. Phys..

[98] Küchle W., Dolg M., Stoll H., Preuss H. (1994). Energy-adjusted pseudopotentials for the actinides. Parameter sets and test calculations for thorium and thorium monoxide. J. Chem. Phys..

[99] Andrae D., Haeussermann U., Dolg M., Stoll H., Preuss H. (1990). Energy-adjusted ab initio pseudopotentials for the second and third row transition elements. Theor. Chim. Acta.

[100] Hariharan P.H., Pople J.A. (1973). The Influence of Polarization Functions on Molecular Orbital Hydrogenation Energies. Theor. Chim. Acta.

[101] Perdew J.P., Burke K., Enzerhof M. (1996). Generalized Gradient Approximation Made Simple. Phys. Rev. Lett..

[102] O’Boyle N.M., Tenderholt A.L., Langner K.M. (2008). cclib: A library for package-independent computational chemistry algorithms. J. Comput. Chem..

[103] Hanwell M.D., Curtis D.E., Lonie D.C., Vandermeersch T., Zurek E., Hutchison G.R. (2012). Avogadro: An advanced semantic chemical editor, visualization, and analysis platform. J. Cheminform..

[104] Tange O. (2018). GNU Parallel 2018. https://www.gnu.org/software/parallel/.

[105] Humphrey W., Dalke A., Schulten K. (1996). VMD: Visual molecular dynamics. J. Mol. Graph..

[106] (2004). Persistence of Vision Pty. Ltd.. Persistence of Vision Raytracer (Version 3.7).

[107] Stoll S., Schweiger A. (2006). EasySpin, a comprehensive software package for spectral simulation and analysis in EPR. J. Magn. Res..

[108] Sheldrick G. (2015). Crystal structure refinement with SHELXL. Acta Cryst. C.

