# Supplementary material for: A “Pretender” Croconate-Bridged Macrocyclic Tetraruthenium Complex: Sizable Redox Potential Splittings despite Electronically Insulated Divinylphenylene Diruthenium Entities
Source: Molecules. 2021 Aug 29;26(17):5232. doi: 10.3390/molecules26175232 (PMC8433806; doi:10.3390/molecules26175232)
Supplement: Supplementary file 1 [file molecules-26-05232-s001.zip › molecules-1336628-supplementary.pdf]

Supplementary Materials

A ”Pretender“ Croconate-Bridged Macrocyclic Tetraruthenium Complex: Sizable Redox Potential Splittings Despite Electronically Insulated Divinylphenylene Diruthenium Entities

Nils Rotthowe[1,2], Michael Linseis[1], Lars Vogelsang[1], Nicole Orth,[3] Ivana Ivanović-Burmazović,[3,4] and Rainer F. Winter[1,*]

[1] Fachbereich Chemie der Universität Konstanz, Universitätsstraße 31, 78464 Konstanz, Germany; nils.rotthowe@uni-konstanz.de (N.R.); michael.linseis@uni-konstanz.de (M- L.); lars.vogelsang@uni-konstanz.de (L.V.)

[2] Present Address: Department of Chemistry, University of Southern California, LJS 251, 840 Downey Way, Los Angeles, CA 90089USA

[3] Department Chemie und Pharmazie, Friedrich-Alexander-Universität Erlangen-Nürnberg, Egerlandstraße 1, 91058 Erlangen, Germany

[4] Department Chemie, Ludwigs-Maximilians-Universität München, Butenandtstr. 5-13, Haus D, 81377 München, Germany

*Correspondence: rainer.winter@uni-konstanz.de; Tel. +49 (7531) 88-5355

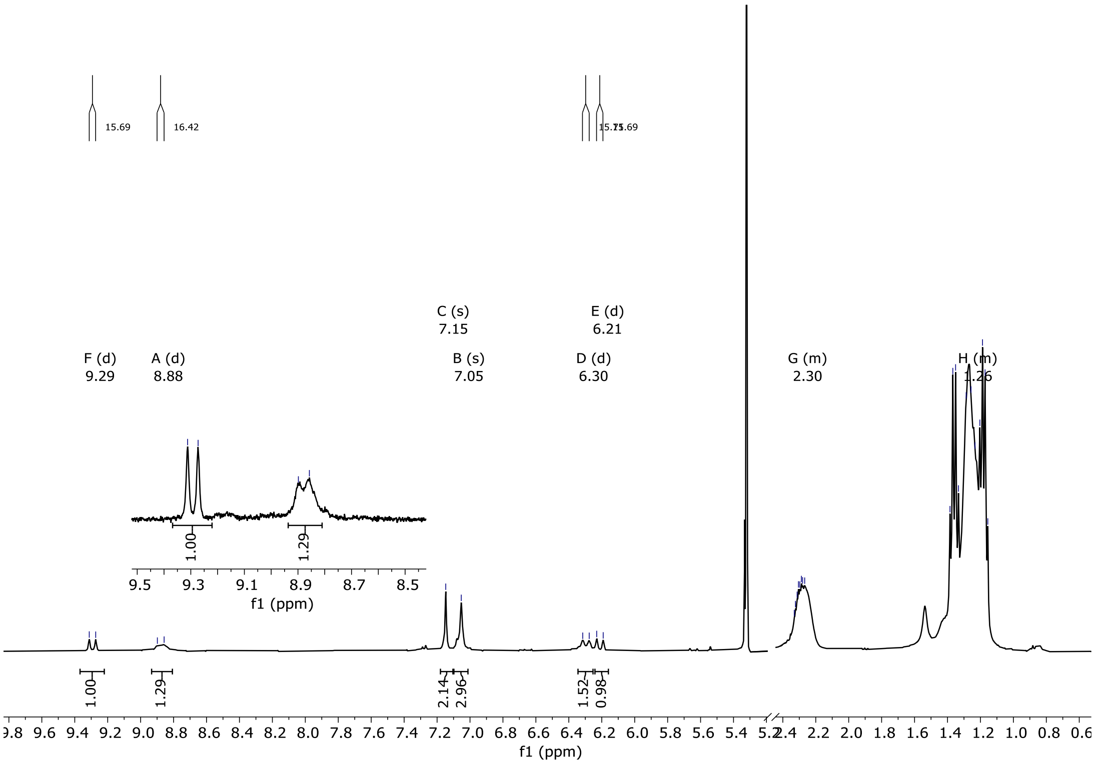

Figure S1. ^{1}H-NMR (400 MHz) spectrum at 300 K of a mixture of **^{2}Ru$_2$Ph-Croc** and **^{4}Ru$_2$Ph-Croc** in CD_2Cl_2.

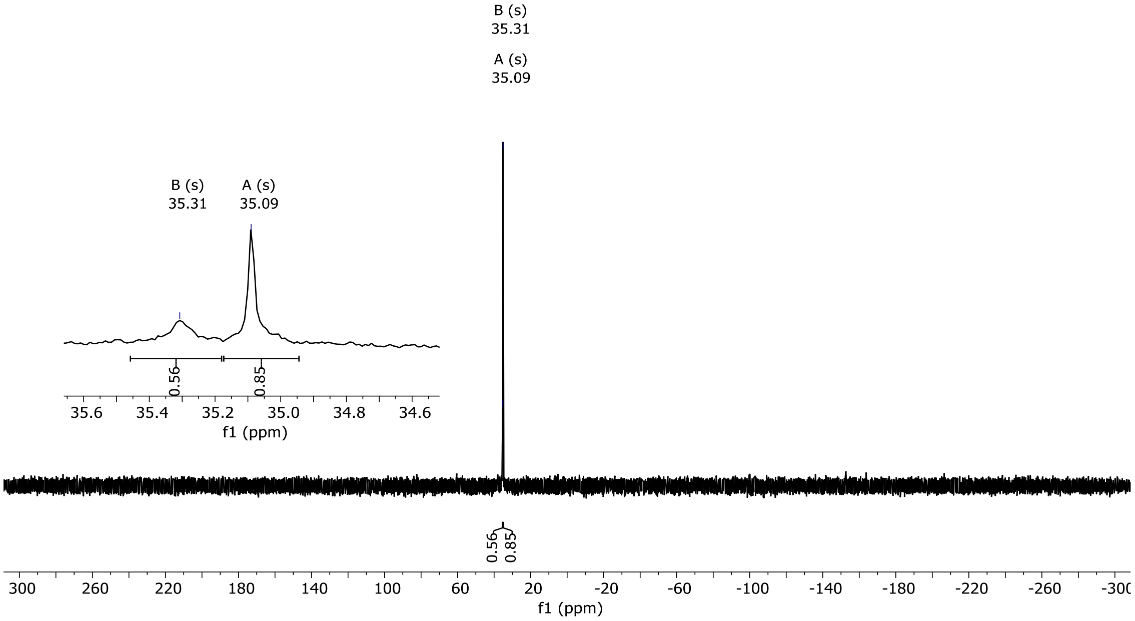

Figure S2. ^{31}P{^{1}H}-NMR (162 MHz) spectrum at 300 K of a mixture of **^{2}Ru$_2$Ph-Croc** and **^{4}Ru$_2$Ph-Croc** in CD_2Cl_2.

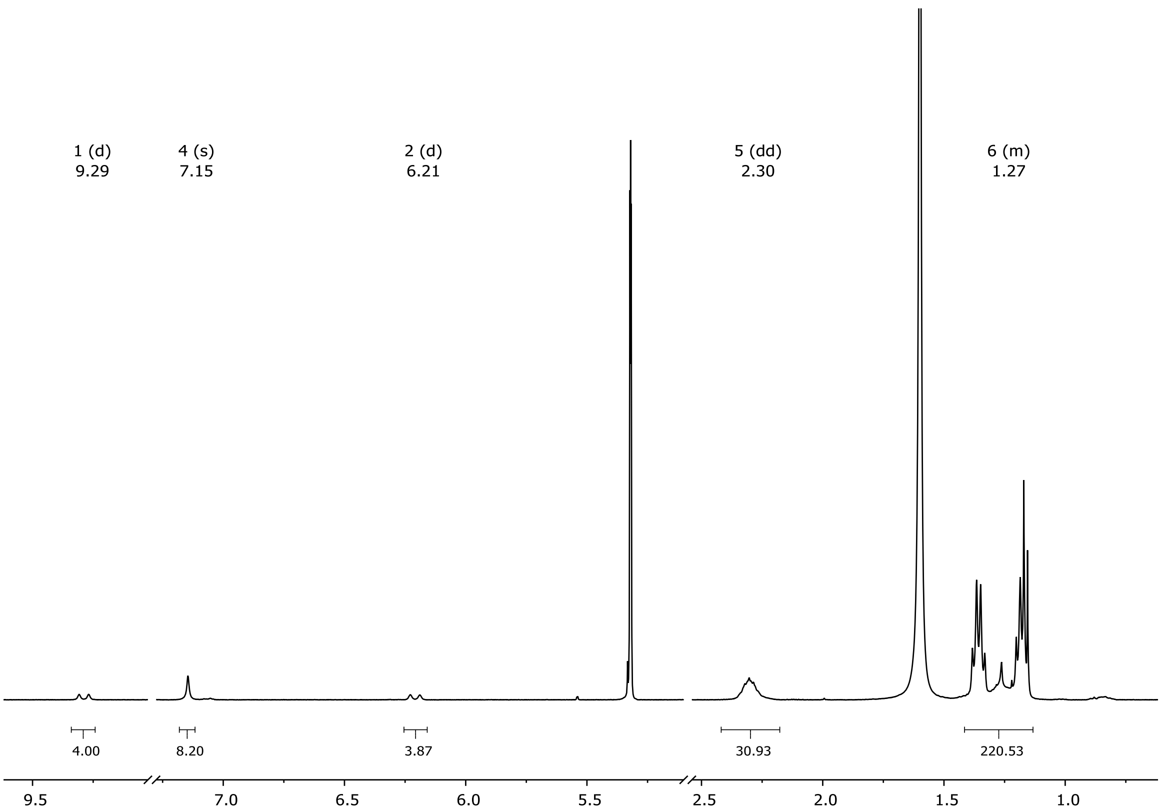

Figure S3. ^{1}H-NMR (800 MHz) spectrum at 300 K of **^{2}Ru$_2$Ph-Croc** in CD_2Cl_2.

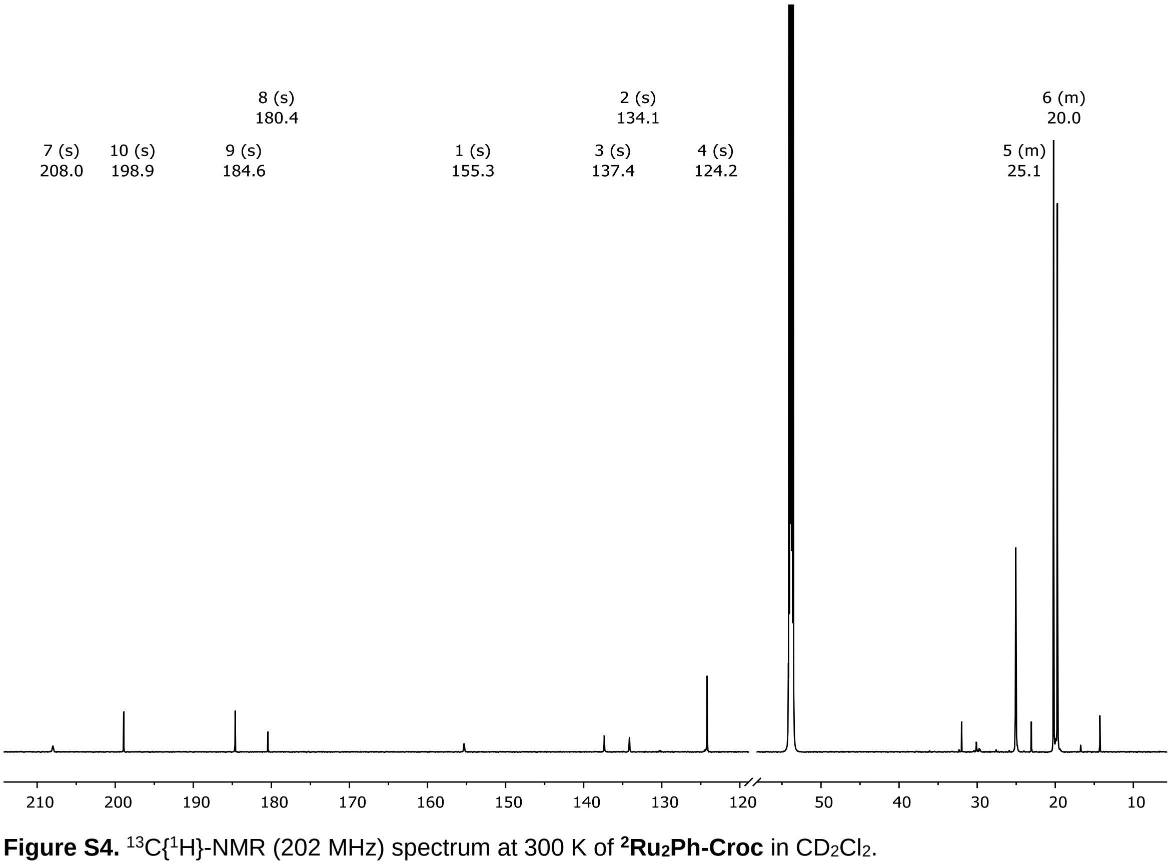

Figure S4. $^{13}C\{^{1}H\}$-NMR (202 MHz) spectrum at 300 K of **^{2}Ru$_2$Ph-Croc** in CD_2Cl_2.

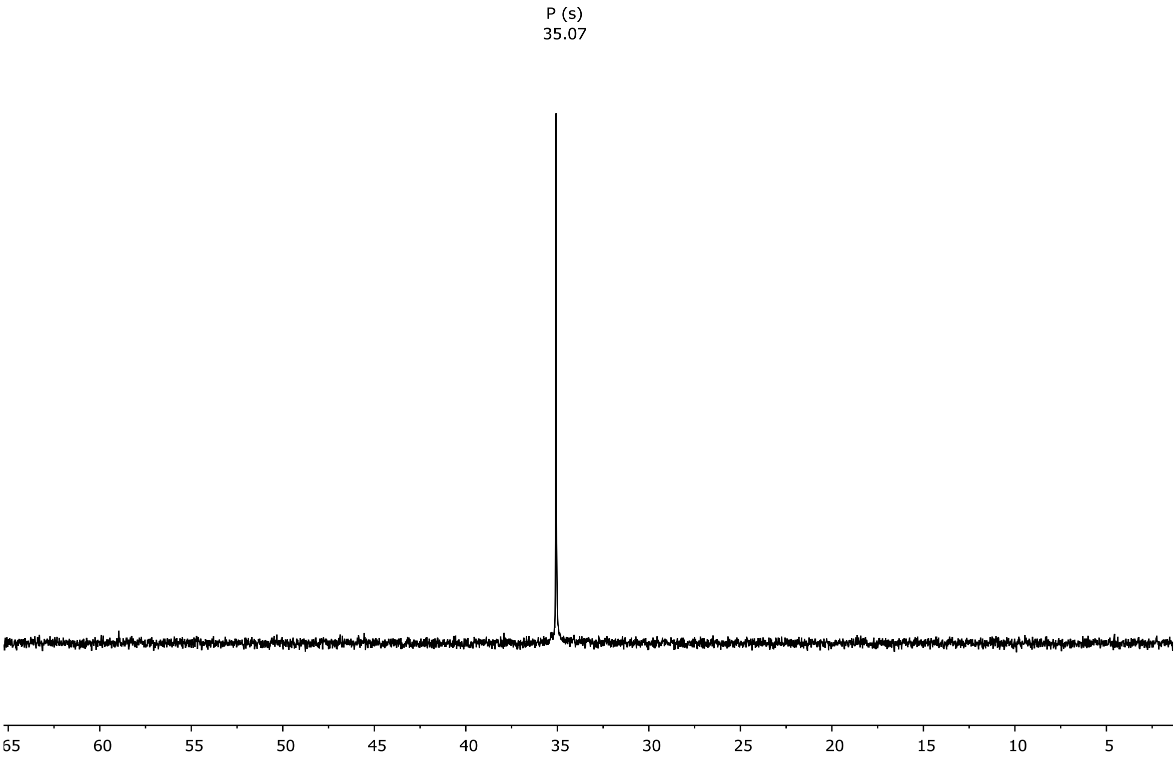

Figure S5. $^{31}P\{^1H\}$-NMR (162 MHz) spectrum at 300 K of **^{2}Ru$_2$Ph-Croc** in CD_2Cl_2.

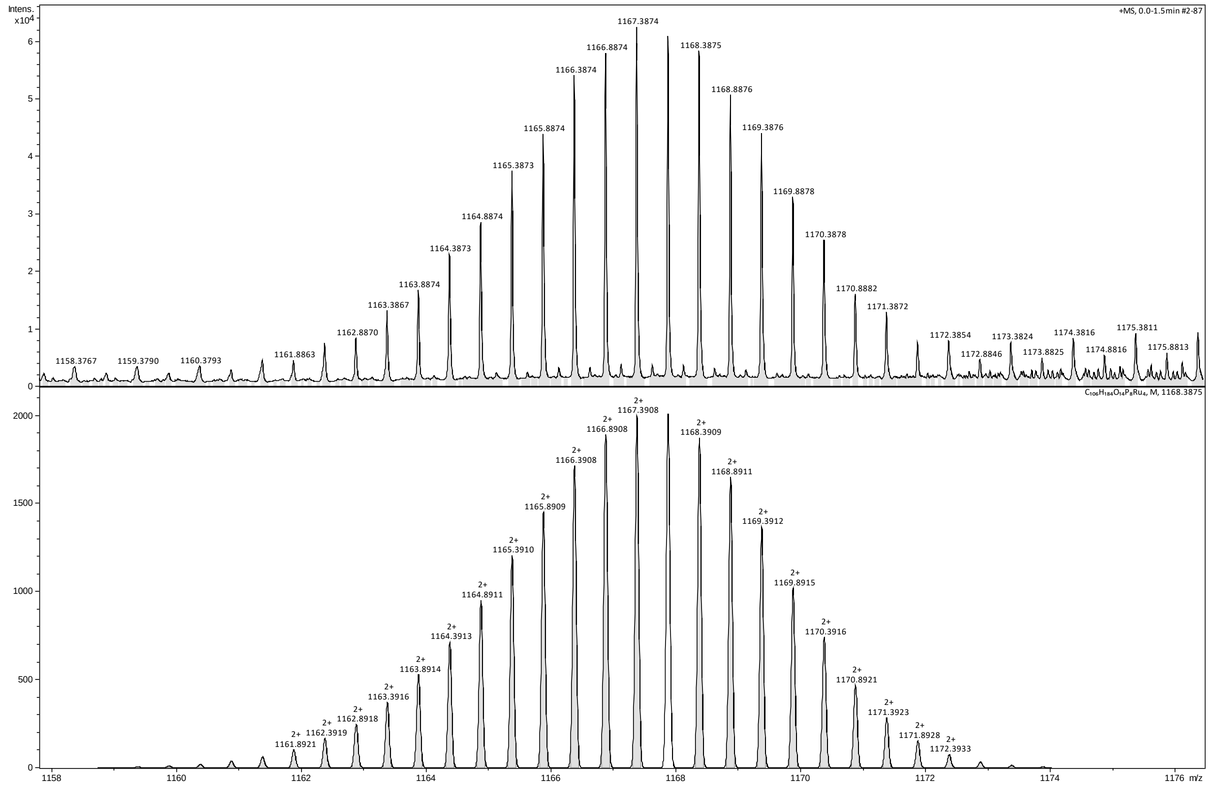

Figure S6. High-resolution ESI [+] mass spectrum of **^{2}Ru$_2$Ph-Croc^{2+}** (top) with corresponding simulation (bottom).

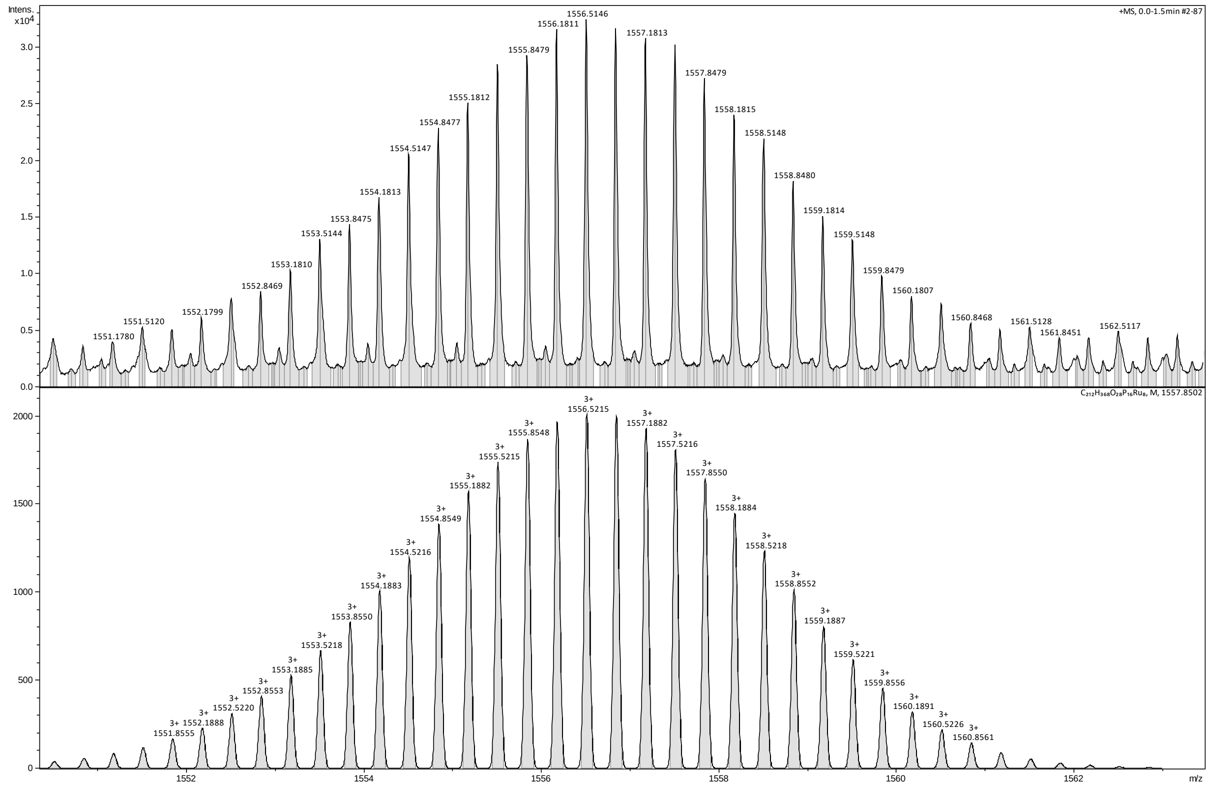

Figure S7. High-resolution ESI [+] mass spectrum of ^{4}**Ru$_2$Ph-Croc^{3+}** (top) with corresponding simulation (bottom).

Table S1. Crystal data and structure refinement for **$^{2}Ru_2$Ph-Croc**.

Identification code	**$^{2}Ru_2$Ph-Croc**
Empirical formula	$C_{53}H_{92}O_7P_4Ru_2$ [+ 1 CH_2Cl_2, 1 CH_3OH]
Formula weight	1167.28
Temperature/K	100
Crystal system	monoclinic
Space group	$P2_1/n$
a/Å	15.1728(11)
b/Å	18.3476(13)
c/Å	22.9663(17)
α/°	90
β/°	104.473(6)
γ/°	90
Volume/Å^3	6190.6(8)
Z	4
ρ_{calc}g/cm^3	1.252
μ/mm^{-1}	0.634
F(000)	2456.0
Crystal size/mm^3	0.5 × 0.333 × 0.2
Radiation	MoKα (λ = 0.71073)
2Θ range for data collection/°	4.284 to 54.032
Index ranges	$-17 \le h \le 19$, $-23 \le k \le 23$, $-29 \le l \le 29$
Reflections collected	37444
Independent reflections	13529 [R_{int} = 0.0540, R_{sigma} = 0.0605]
Data/restraints/parameters	13529/0/620
Goodness-of-fit on F^2	1.091
Final R indexes [I>=2σ (I)]	R_1 = 0.0683, wR_2 = 0.1861
Final R indexes [all data]	R_1 = 0.1027, wR_2 = 0.2120
Largest diff. peak/hole / e Å^{-3}	1.46/-1.10

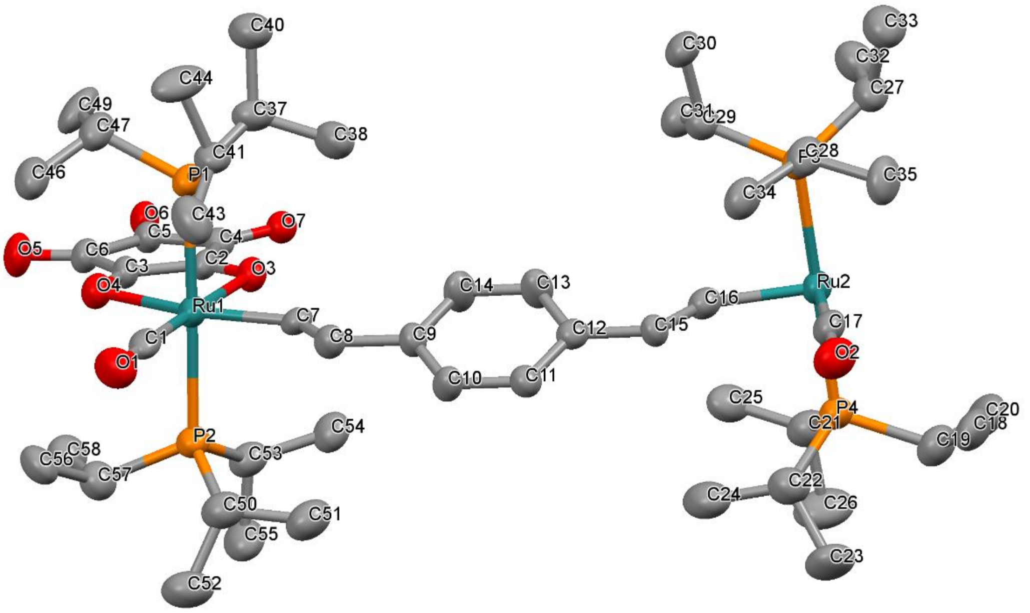

Figure S8. Asymmetric unit of [2]**Ru_2Ph-Croc** with atom labels.

Table S2. Bond lengths for [2]**Ru$_2$Ph-Croc**.

Atom	Atom	Length/Å	Atom	Atom	Length/Å
Ru1	P2	2.4261(14)	C3	C4	1.408(7)
Ru1	P1	2.4126(15)	C17	C13	1.414(8)
Ru1	O7[1]	2.202(4)	C14	C13	1.446(7)
Ru1	O61	2.302(3)	C14	C15	1.497(8)
Ru1	C1	2.026(5)	C2	C1	1.340(7)
Ru1	C11	1.803(7)	C8	C7	1.400(7)
Ru2	P4	2.4480(14)	C30	C32	1.530(7)
Ru2	P3	2.4226(14)	C30	C31	1.532(8)
Ru2	O3	2.202(4)	C6	C7	1.390(7)
Ru2	O4	2.292(4)	C6	C9	1.491(7)
Ru2	C10	2.026(5)	C6	C5	1.404(8)
Ru2	C12	1.813(6)	C39	C41	1.552(8)
P2	C30	1.874(6)	C39	C40	1.542(8)
P2	C33	1.863(6)	C10	C9	1.346(8)
P2	C27	1.859(6)	C21	C23	1.526(9)
P4	C51	1.858(7)	C21	C22	1.545(9)
P4	C48	1.891(8)	C33	C34	1.489(10)
P4	C45	1.878(8)	C33	C35	1.542(9)
P3	C39	1.853(6)	C5	C4	1.386(8)
P3	C36	1.893(7)	C29	C27	1.540(9)
P3	C42	1.876(6)	C27	C28	1.558(8)
P1	C21	1.852(7)	C43	C42	1.550(9)
P1	C18	1.875(6)	C44	C42	1.543(9)
P1	C24	1.884(7)	C36	C37	1.551(9)
O3	C13	1.282(6)	C36	C38	1.511(9)
O7	C17	1.285(6)	C18	C20	1.543(10)
O4	C14	1.248(6)	C18	C19	1.544(9)
O6	C16	1.256(6)	C24	C25	1.533(9)
O5	C15	1.229(6)	C24	C26	1.520(10)
O2	C12	1.175(7)	C49	C48	1.505(10)
O1	C11	1.170(7)	C51	C52	1.500(11)
C16	C17	1.457(7)	C51	C53	1.539(11)
C16	C15	1.498(7)	C46	C45	1.489(11)
C3	C2	1.471(7)	C48	C50	1.560(10)
C3	C8	1.395(7)	C45	C47	1.555(11)

[1]1-X,1-Y,1-Z

Table S3. Bond angles for ^{2}**Ru$_2$Ph-Croc**.

Atom	Atom	Atom	Angle/°	Atom	Atom	Atom	Angle/°
P1	Ru1	P2	177.55(6)	C4	C3	C2	121.7(5)
O7^1	Ru1	P2	91.97(10)	O7	C17	C16	120.3(5)
O7^1	Ru1	P1	90.46(10)	O7	C17	C13	131.0(5)
O7^1	Ru1	O6^1	75.89(13)	C13	C17	C16	108.7(4)
O6^1	Ru1	P2	91.38(10)	O4	C14	C13	122.7(5)
O6^1	Ru1	P1	89.44(10)	O4	C14	C15	128.4(5)
C1	Ru1	P2	88.12(14)	C13	C14	C15	109.0(5)
C1	Ru1	P1	91.77(14)	O3	C13	C17	129.9(5)
C1	Ru1	O7^1	87.09(18)	O3	C13	C14	120.6(5)
C1	Ru1	O6^1	162.96(19)	C17	C13	C14	109.4(5)
C11	Ru1	P2	91.5(2)	C1	C2	C3	124.7(5)
C11	Ru1	P1	86.1(2)	C3	C8	C7	122.6(5)
C11	Ru1	O7^1	175.7(2)	O5	C15	C16	127.5(5)
C11	Ru1	O6^1	106.7(2)	O5	C15	C14	128.4(5)
C11	Ru1	C1	90.4(2)	C14	C15	C16	104.1(4)
P3	Ru2	P4	178.68(5)	C32	C30	P2	116.1(4)
O3	Ru2	P4	88.35(10)	C32	C30	C31	110.2(5)
O3	Ru2	P3	92.32(10)	C31	C30	P2	114.5(4)
O3	Ru2	O4	76.20(13)	C7	C6	C9	121.4(5)
O4	Ru2	P4	91.15(10)	C7	C6	C5	116.4(5)
O4	Ru2	P3	87.91(10)	C5	C6	C9	122.2(5)
C10	Ru2	P4	89.44(14)	C2	C1	Ru1	138.1(4)
C10	Ru2	P3	91.74(14)	C41	C39	P3	114.7(4)
C10	Ru2	O3	85.61(19)	C40	C39	P3	115.3(5)
C10	Ru2	O4	161.77(19)	C40	C39	C41	110.1(5)
C12	Ru2	P4	93.20(17)	C6	C7	C8	121.3(5)
C12	Ru2	P3	86.14(17)	C9	C10	Ru2	140.4(4)
C12	Ru2	O3	178.36(19)	O2	C12	Ru2	177.7(5)
C12	Ru2	O4	104.3(2)	C10	C9	C6	123.8(5)
C12	Ru2	C10	93.9(3)	C23	C21	P1	115.0(5)
C30	P2	Ru1	113.96(17)	C23	C21	C22	111.7(6)
C33	P2	Ru1	113.1(2)	C22	C21	P1	114.5(5)
C33	P2	C30	108.8(3)	O1	C11	Ru1	175.5(6)
C27	P2	Ru1	115.2(2)	C34	C33	P2	113.9(4)
C27	P2	C30	102.1(3)	C34	C33	C35	108.2(6)
C27	P2	C33	102.6(3)	C35	C33	P2	115.7(5)
C51	P4	Ru2	115.4(2)	C4	C5	C6	122.4(5)
C51	P4	C48	104.0(3)	C5	C4	C3	121.4(5)
C51	P4	C45	101.0(4)	C29	C27	P2	113.5(4)
C48	P4	Ru2	118.1(2)	C29	C27	C28	111.2(5)
C45	P4	Ru2	114.3(2)	C28	C27	P2	111.8(4)
C45	P4	C48	101.8(4)	C37	C36	P3	117.1(5)
C39	P3	Ru2	114.1(2)	C38	C36	P3	113.5(5)

C39	P3	C36	109.2(3)	C38	C36	C37	108.9(6)
C39	P3	C42	102.1(3)	C43	C42	P3	115.8(5)
C36	P3	Ru2	114.1(2)	C44	C42	P3	111.7(4)
C42	P3	Ru2	114.5(2)	C44	C42	C43	109.7(5)
C42	P3	C36	101.5(3)	C20	C18	P1	110.4(5)
C21	P1	Ru1	115.1(2)	C20	C18	C19	110.6(6)
C21	P1	C18	102.3(3)	C19	C18	P1	115.4(5)
C21	P1	C24	108.8(3)	C25	C24	P1	116.4(5)
C18	P1	Ru1	114.7(2)	C26	C24	P1	114.4(5)
C18	P1	C24	102.4(3)	C26	C24	C25	109.2(6)
C24	P1	Ru1	112.4(2)	C52	C51	P4	116.3(5)
C13	O3	Ru2	111.5(3)	C52	C51	C53	109.3(7)
C17	O7	Ru1[1]	112.2(3)	C53	C51	P4	109.9(5)
C14	O4	Ru2	108.8(3)	C49	C48	P4	114.4(5)
C16	O6	Ru1[1]	109.2(3)	C49	C48	C50	105.6(7)
O6	C16	C17	122.0(4)	C50	C48	P4	116.9(5)
O6	C16	C15	129.2(5)	C46	C45	P4	113.8(5)
C17	C16	C15	108.8(5)	C46	C45	C47	109.0(7)
C8	C3	C2	122.3(5)	C47	C45	P4	116.3(6)
C8	C3	C4	115.9(5)				

[1]1-X,1-Y,1-Z

Table S4. Solvent masks information for [2]**Ru$_2$Ph-Croc**.

A solvent mask was calculated and 242 electrons were found in a volume of 886 Å^3 in 1 void per unit cell. This is consistent with the presence of 1 [CH_2Cl_2], 1[CH_4O] per Asymmetric Unit which account for 240 electrons per unit cell.

Number	X	Y	Z	Volume	Electron count	Content
1	0.000	0.000	0.000	443.0	120.5	2 CH_2Cl_2, 2 MeOH
2	0.500	0.500	0.500	443.0	120.5	2 CH_2Cl_2, 2 MeOH

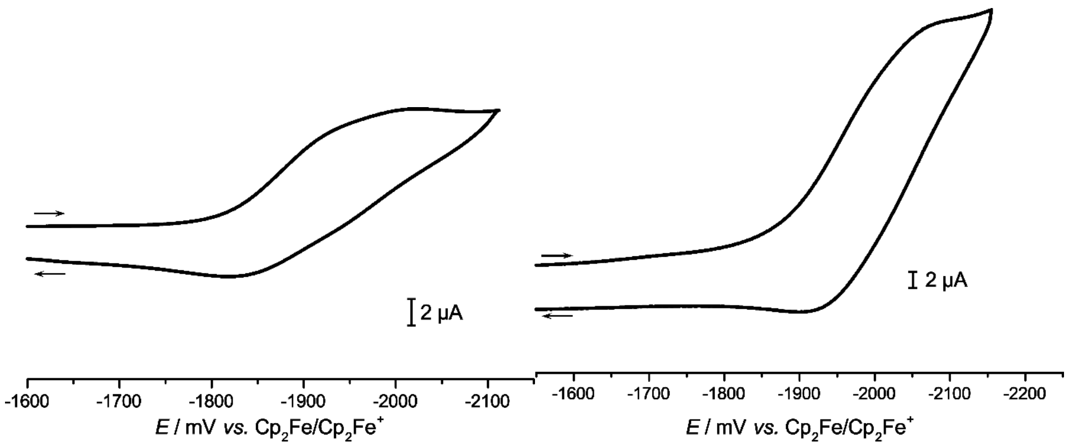

Figure S9. Cathodic scans in the cyclic voltammograms of **^{2}Ru$_2$Ph-Croc** in CH_2Cl_2 with either 0.1 M NBu_4PF_6 (left panel) or $NBu_4BAr^F_{24}$ (right panel) as the supporting electrolyte.

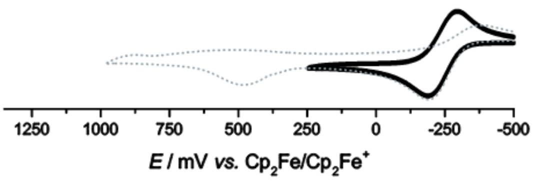

Figure S10. Anodic scan in the cyclic voltammogram of **$(NBu_4^+)_2$ croc^{2-}** in CH_2Cl_2 with 0.1 M NBu_4PF_6 as the supporting electrolyte.

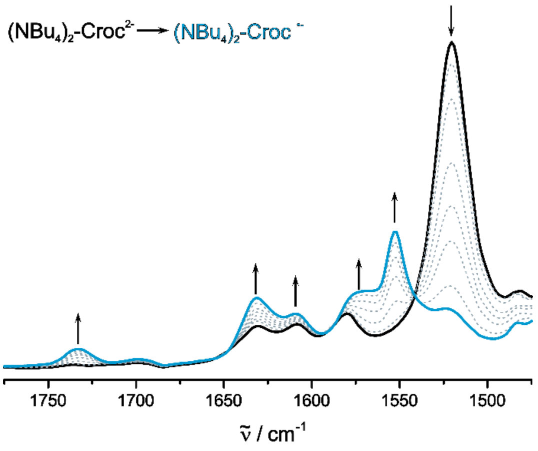

Figure S11. Changes of the IR spectra (DCE, 0.25 M NBu_4PF_6, r. t.) of **$(NBu_4^+)_2$ croc^{2-}** in the C=O/C=C stretching region during the first oxidation to the radical anion.

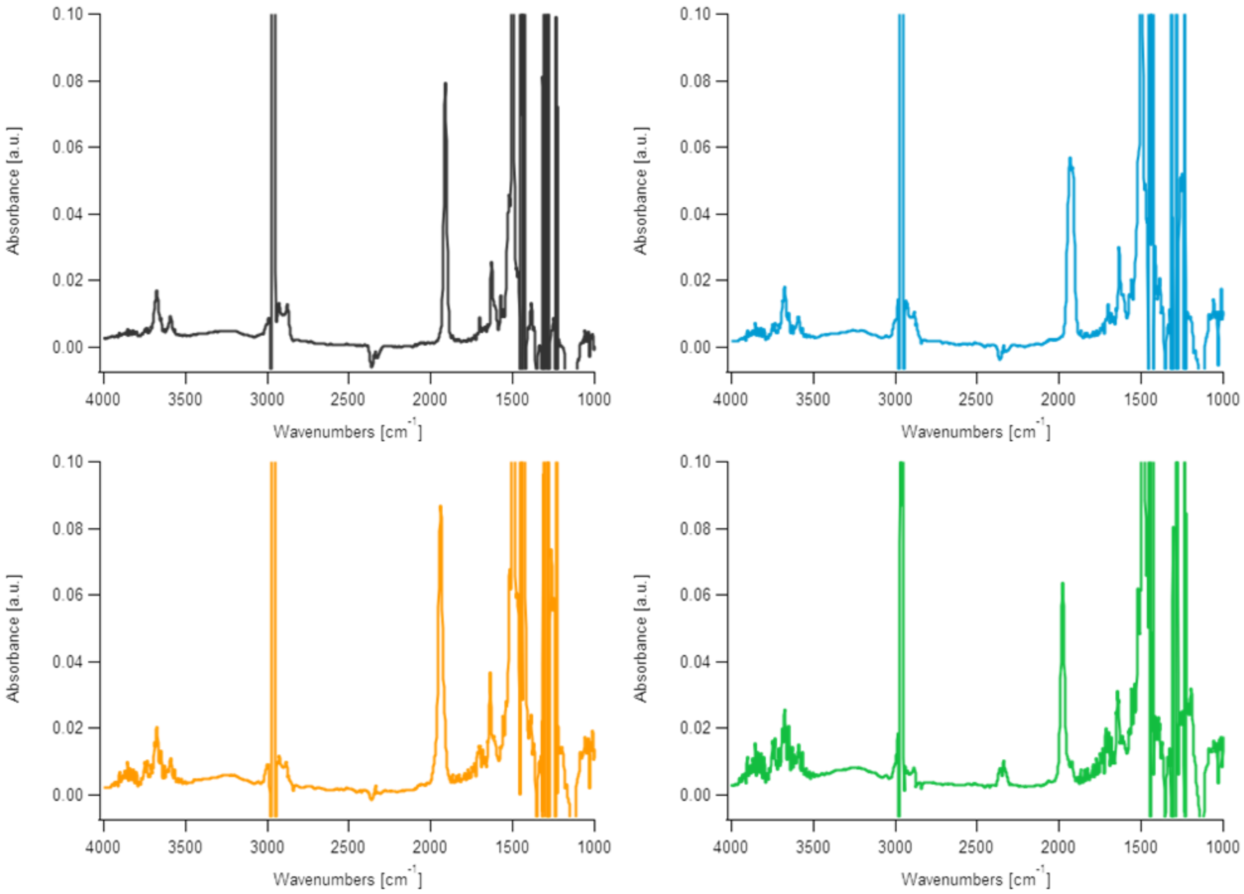

Figure S12. IR spectra of **^{2}Ru$_2$Ph-Croc** in the neutral (top, left), monocationic (top, right), dicationic (bottom, left) and tetracationic (bottom, right) state as obtained during the spectroelectrochemical investigation in CH_2Cl_2/NBu_4PF_6. Here the data for the measurement containing the PF_6^- counterion is presented to prevent contamination with absorbance bands originating from the $BAr^F{}_{24}^-$ anion.

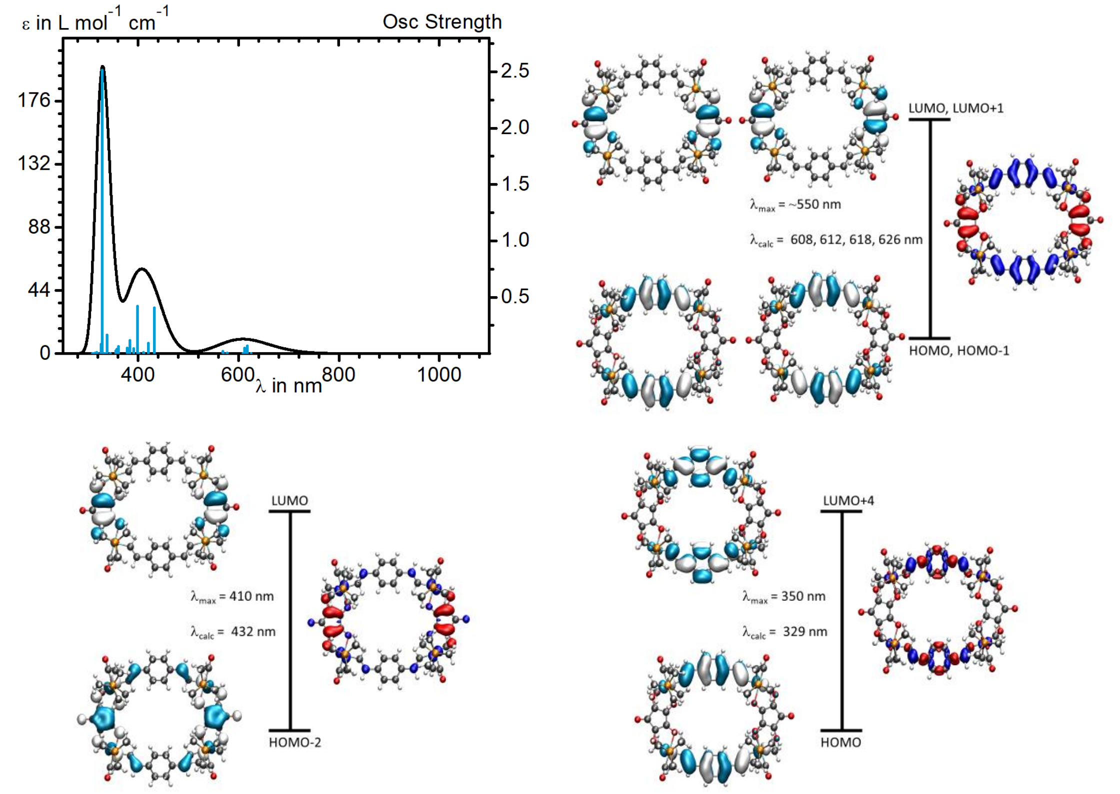

Figure S13. TD-DFT calculated UV/vis/NIR absorption spectrum of neutral **^{2}Ru$_2$Ph-Croc** and graphical representation of the MOs with the largest contribution to the relevant transitions as well as changes in electron density distributions accompanying the electronic excitations (blue: decreasing electron density; red: increasing electron density).

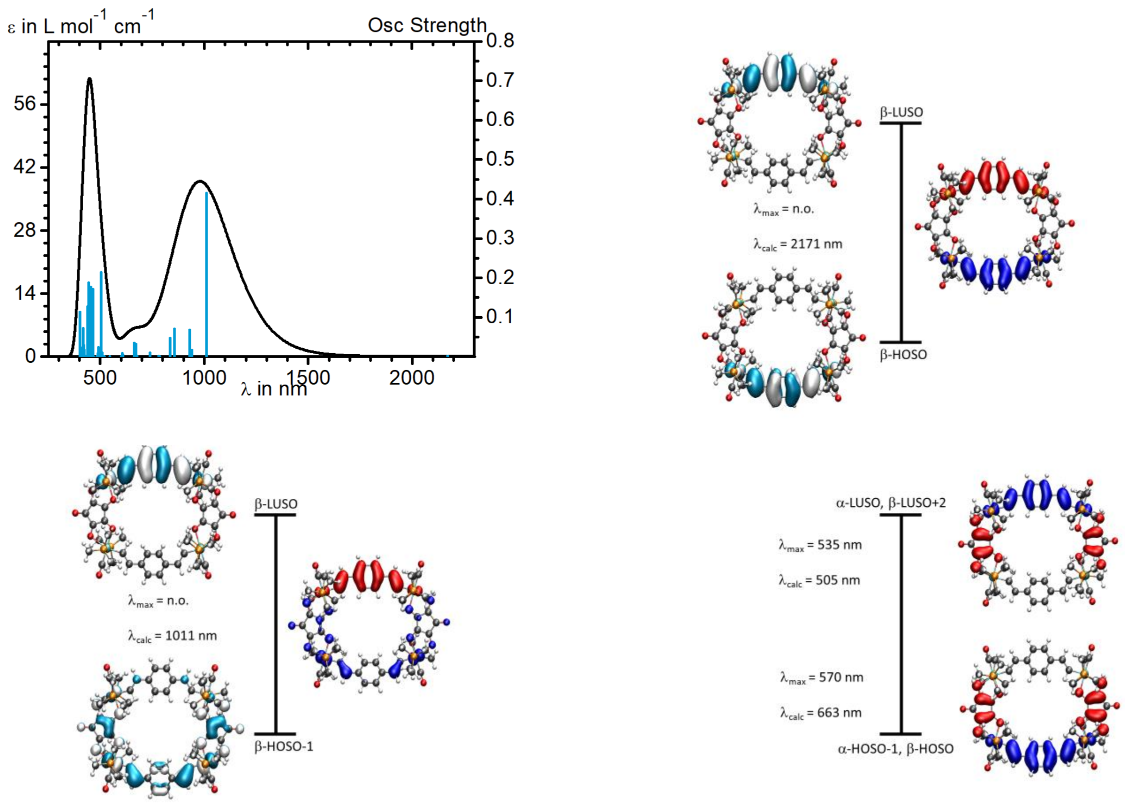

Figure S14. TD-DFT calculated UV/Vis/NIR absorption spectrum of the one-electron oxidized form **^{2}Ru$_2$Ph-Croc$^{•+}$** and graphical representations of MOs with the largest contribution to the relevant transitions along with the according electron density difference maps (blue: decreasing electron density; red: increasing electron density).

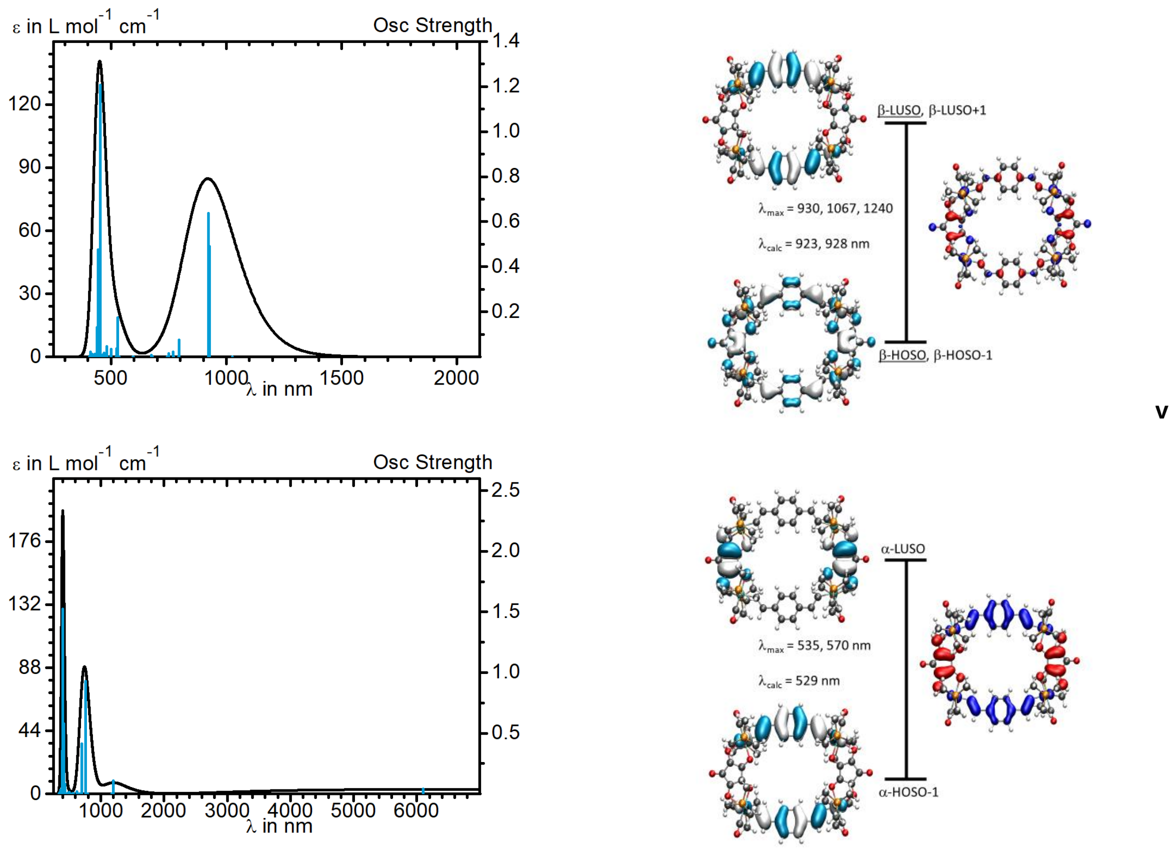

Figure S15. TD-DFT calculated UV/Vis/NIR absorption spectrum of dicationic **^{2}Ru$_2$Ph-Croc^{2+}** in the triplet (top left) and singlet states (bottom left) along with graphical representations of MOs with the largest contribution to the relevant transitions and electron density difference maps (blue: decreasing electron density; red: increasing electron density).

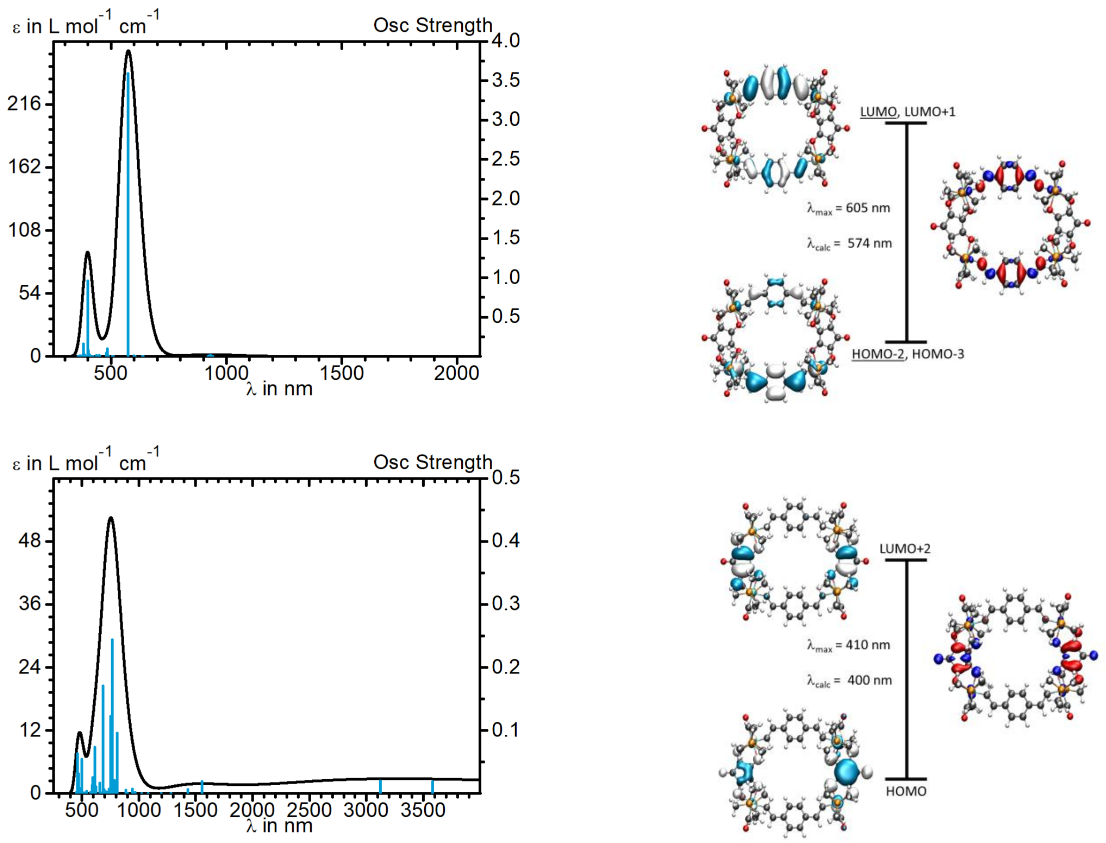

Figure S16. TD-DFT calculated UV/Vis/NIR absorption spectrum of tetracationic ^{2}**Ru$_2$Ph-Croc^{4+}** in the singlet (top left) and quintet state (bottom left) along with graphical representations of MOs with the largest contribution to the relevant transitions and electron density difference maps (blue: decreasing electron density; red: increasing electron density).

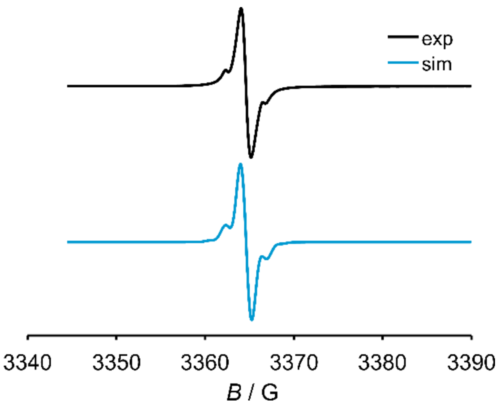

Figure S17. Experimental (top curve) and simulated (bottom curve) EPR spectrum of **NBu$_4$Croc$^{\bullet-}$** at +20 °C in CH_2Cl_2 solution.

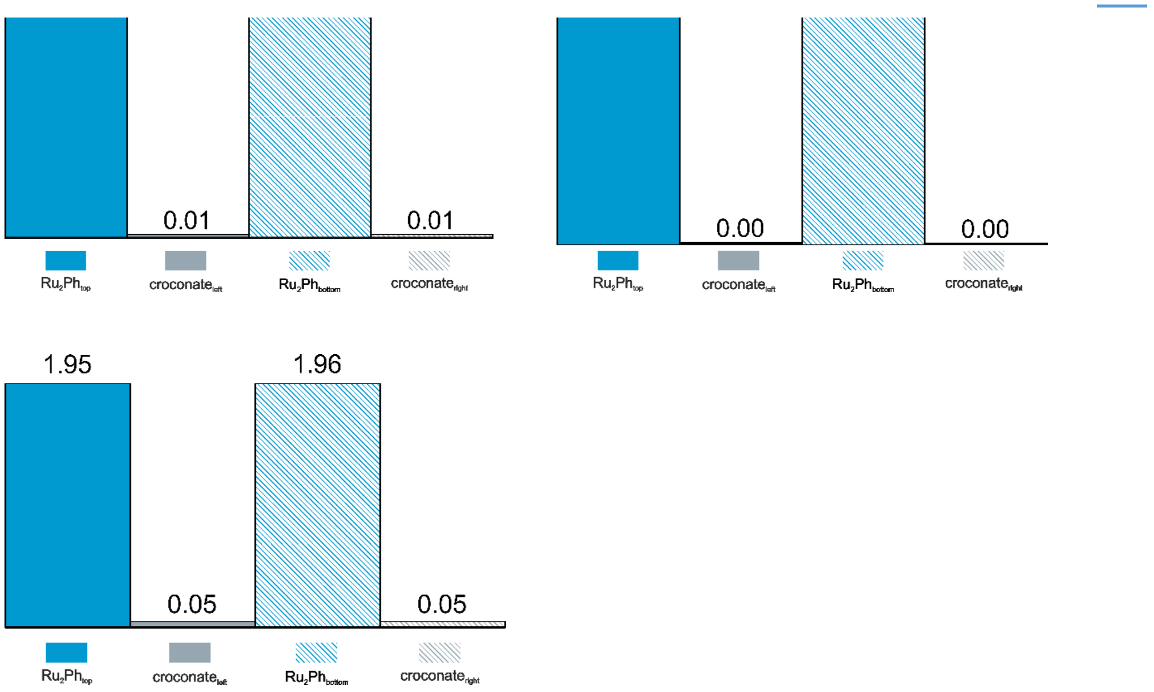

Figure S18. Calculated MULLIKEN spin densities on different parts of ^{2}**Ru$_2$Ph-Croc$^{\bullet+}$** (top left), ^{2}**Ru$_2$Ph-Croc^{2+}** in the triplet state (top right), and ^{2}**Ru$_2$Ph-Croc^{4+}** (bottom left)

Table S 5. Input file for the geometry optimization and frequency analysis of $^{2}Ru_2$Ph-Croc in Gaussian16

```
%chk=pMaurer_croc_4_n_pbe1pbe_DCM.chk
%mem=28000MB
%nprocshared=10
# opt freq pbe1pbe/Gen Pseudo=Read scrf=(PCM,solvent=CH2CL2,read)

pMaurer_croc_4_n_pbe1pbe_DCM

0 1
 Ru     9.82040000    5.01940000   11.77450000
 Ru     5.56250000   13.93300000    6.36330000
 P     10.96980000    6.57580000   13.23730000
 P      6.58800000   15.31000000    8.10820000
 P      4.53660000   12.61950000    4.60580000
 P      8.76760000    3.45490000   10.26930000
 O      4.13300000   13.20590000    7.86560000
 O      1.55440000   12.88590000    9.53190000
 O      3.80000000   15.38720000    6.18690000
 O     -0.20640000   14.87660000    8.77070000
 O      1.07590000   16.63940000    6.65370000
 O      7.49640000   14.81390000    4.25780000
 O     12.28840000    4.36860000   10.24040000
 C      0.92220000   14.77350000    8.23680000
 C      9.49000000    8.32610000    8.75490000
 C      1.86070000   13.73320000    8.62150000
 C      2.89860000   15.03590000    6.96920000
 C      3.04930000   13.88550000    7.84320000
 C     10.04190000    7.15370000    9.46200000
 H     10.84540000    6.79820000    9.15630000
 C      9.89410000    8.64910000    7.45400000
 H     10.52090000    8.10930000    7.02990000
 C      1.54760000   15.63770000    7.18940000
 C     10.39850000    8.34820000   13.05340000
 H     10.52700000    8.56110000   12.10480000
 C      8.43350000   10.60310000    7.36950000
 C      9.45780000    6.57030000   10.51390000
 H      8.65930000    6.99630000   10.72860000
 C      3.21440000   11.45810000    5.19910000
 H      2.54030000   12.03100000    5.61980000
 C      9.37940000    9.76090000    6.77350000
 H      9.67360000    9.94130000    5.90970000
 C      6.72260000   12.36810000    6.93810000
 H      6.30240000   11.89850000    7.62240000
 C      6.73110000   14.49830000    5.09460000
 C      7.89160000   11.79570000    6.65350000
 H      8.40060000   12.16120000    5.96580000
 C      6.90830000    3.53190000   10.21590000
 H      6.61840000    3.27940000   11.11710000
 C     11.34940000    4.63460000   10.89190000
 C     10.87940000    6.09140000   15.03480000
 H     10.92710000    5.11360000   15.05490000
 C      8.03400000   10.27830000    8.67040000
 H      7.40840000   10.81840000    9.09720000
 C      8.53720000    9.17560000    9.34860000
 H      8.24030000    8.99390000   10.21030000
 C     12.80290000    6.71890000   12.95560000
 H     13.14320000    7.33040000   13.64270000
 C      5.76630000   11.65810000    3.52460000
 H      6.58660000   12.19560000    3.52700000
 C      3.62120000   13.62310000    3.30000000
 H      3.45340000   13.02220000    2.54480000
 C      9.08080000    1.64390000   10.66950000
 H      8.84280000    1.13200000    9.86940000
 C      9.42060000    3.57960000    8.51250000
 H     10.34660000    3.88470000    8.61100000
```

C	6.10970000	17.11830000	8.10780000
H	6.72540000	17.58580000	8.71060000
C	8.49330000	15.34410000	8.20790000
H	8.75790000	14.58640000	8.77000000
C	6.11290000	14.81200000	9.86450000
H	5.13650000	14.89450000	9.90130000
Ru	-0.38740000	13.32820000	10.46300000
Ru	3.87050000	4.41460000	15.87420000
P	-1.53680000	11.77180000	9.00020000
P	2.84500000	3.03760000	14.12920000
P	4.89640000	5.72810000	17.63170000
P	0.66530000	14.89270000	11.96820000
O	5.30000000	5.14170000	14.37190000
O	7.87860000	5.46170000	12.70560000
O	5.63290000	2.96040000	16.05060000
O	9.63940000	3.47100000	13.46680000
O	8.35710000	1.70820000	15.58380000
O	1.93660000	3.53370000	17.97970000
O	-2.85540000	13.97900000	11.99710000
C	8.51080000	3.57410000	14.00070000
C	-0.05710000	10.02150000	13.48260000
C	7.57230000	4.61440000	13.61600000
C	6.53430000	3.31170000	15.26830000
C	6.38360000	4.46210000	14.39430000
C	-0.60890000	11.19390000	12.77540000
H	-1.41240000	11.54940000	13.08120000
C	-0.46110000	9.69850000	14.78350000
H	-1.08790000	10.23830000	15.20760000
C	7.88540000	2.70990000	15.04810000
C	-0.96550000	9.99940000	9.18410000
H	-1.09410000	9.78650000	10.13270000
C	0.99940000	7.74450000	14.86800000
C	-0.02490000	11.77730000	11.72360000
H	0.77360000	11.35130000	11.50890000
C	6.21860000	6.88950000	17.03840000
H	6.89270000	6.31660000	16.61760000
C	0.05360000	8.58670000	15.46390000
H	-0.24060000	8.40630000	16.32780000
C	2.71030000	5.97950000	15.29940000
H	3.13060000	6.44910000	14.61510000
C	2.70180000	3.84930000	17.14290000
C	1.54140000	6.55190000	15.58400000
H	1.03240000	6.18640000	16.27160000
C	2.52460000	14.81570000	12.02160000
H	2.81450000	15.06820000	11.12040000
C	-1.91640000	13.71300000	11.34560000
C	-1.44640000	12.25620000	7.20270000
H	-1.49410000	13.23400000	7.18260000
C	1.39890000	8.06930000	13.56710000
H	2.02460000	7.52920000	13.14030000
C	0.89580000	9.17200000	12.88880000
H	1.19270000	9.35370000	12.02720000
C	-3.36990000	11.62870000	9.28190000
H	-3.71020000	11.01720000	8.59480000
C	3.66670000	6.68950000	18.71280000
H	2.84640000	6.15200000	18.71040000
C	5.81180000	4.72450000	18.93740000
H	5.97960000	5.32540000	19.69270000
C	0.35220000	16.70370000	11.56790000
H	0.59020000	17.21560000	12.36810000
C	0.01240000	14.76800000	13.72500000
H	-0.91370000	14.46290000	13.62640000
C	3.32330000	1.22930000	14.12970000
H	2.70760000	0.76180000	13.52690000
C	0.93970000	3.00350000	14.02960000
H	0.67510000	3.76120000	13.46740000

C	3.32000000	3.53560000	12.37290000
H	4.29640000	3.45310000	12.33620000
H	4.22877806	14.42335009	2.93210234
H	2.67198631	14.00944711	3.60761873
H	5.51483190	11.53002785	2.49248503
H	6.04530181	10.71609088	3.94848301
H	2.69110140	10.92861222	4.43052908
H	3.52607877	10.78281901	5.96835414
H	10.12504410	1.48287724	10.83839622
H	8.50270410	1.24240386	11.47541934
H	8.94507646	4.33219199	7.91888171
H	9.49012212	2.66888130	7.95513287
H	6.44570801	2.81639004	9.56863332
H	6.51056942	4.51313720	10.06135567
H	2.95439340	2.90837608	11.58688348
H	3.11283977	4.55727989	12.13174686
H	0.50977389	2.12896638	13.58772391
H	0.43967558	3.13039768	14.96702869
H	3.16937158	0.83916270	15.11407743
H	4.31870402	1.01014478	13.80405936
H	-1.53056710	9.26188869	8.65336821
H	0.07778446	9.84594711	9.00268954
H	-2.24675244	11.90683488	6.58441205
H	-0.52074131	11.99550868	6.73355608
H	-3.87914486	12.55950105	9.14341142
H	-3.61092050	11.19072695	10.22793844
H	3.91816810	6.81757215	19.74491497
H	3.38769093	7.63153365	18.28897629
H	5.90692123	7.56478099	16.26914586
H	6.74189860	7.41898778	17.80697092
H	5.20423545	3.92426771	19.30535870
H	6.76103124	4.33814575	18.62984440
H	11.67975186	6.44079499	15.65307182
H	9.95371409	6.35209899	15.50388595
H	10.96356710	9.08571131	13.58413179
H	9.35521554	8.50165289	13.23481046
H	13.04392050	7.15687305	12.00956156
H	13.31214486	5.78809895	13.09408858
H	-0.05712212	15.67871870	14.28236713
H	0.48794076	14.01538075	14.31856991
H	-0.69204741	16.86471253	11.39901451
H	0.93032426	17.10521583	10.76201082
H	2.98724861	15.53118983	12.66884846
H	2.92239483	13.83447743	12.17607185
H	6.47854991	15.43918070	10.65053086
H	6.32012711	13.79034923	10.10571905
H	8.92316885	16.21865605	8.64978742
H	8.99326810	15.21719833	7.27044180
H	5.11428641	17.33744168	8.43342051
H	6.26362842	17.50843730	7.12342257

H C O P 0
6-31G(d)
****
Ru 0
MWB28
****

Ru 0
MWB28

ModifySph

Ru 2.2

Table-S 6. Input file for the geometry optimization and frequency analysis of the radical cation of $^{2}Ru_2$Ph-Croc in Gaussian16 based on the results produced for the neutral species. The input files for the other oxidation/spin states are of the same format

```
%oldchk=pMaurer_croc_4_n_pbe1pbe_DCM.chk
%chk=pMaurer_croc_4_p1_pbe1pbe_DCM.chk
%mem=28000MB
%nprocshared=10
# opt freq nosymm Geom=Check Guess=Read pbe1pbe/checkbasis pseudo=Read scrf=check

pMaurer_croc_4_p1_pbe1pbe_DCM

1 2
```

Table-S 7. Input file to calculate the first 60 optical transitions of the radical cation of $^{2}Ru_2$Ph-Croc in Gaussian16 via TD-DFT. The input structure was optimized beforehand (vide supra).

```
%oldchk=pMaurer_croc_4_p1_pbe1pbe_DCM.chk
%chk=pMaurer_croc_4_p1_pbe1pbe_DCM_tddft.chk
%mem=28000MB
%nprocshared=10
# Geom=AllCheck Guess=Read pbe1pbe/checkbasis pseudo=Read scrf=check TD=NStates=60
```
